# Cyanido-bridged diplatinum(ii) complexes: ligand and solvent effect on aggregation and luminescence†

**DOI:** 10.1039/d3sc06941a

**Published:** 2024-02-12

**Authors:** Viktoria V. Khistiaeva, Stefan Buss, Toni Eskelinen, Pipsa Hirva, Niko Kinnunen, Joshua Friedel, Lukas Kletsch, Axel Klein, Cristian A. Strassert, Igor O. Koshevoy

**Affiliations:** a Department of Chemistry, University of Eastern Finland P.O. Box 111 FI-80100 Joensuu Finland igor.koshevoy@uef.fi; b Institut für Anorganische und Analytische Chemie, Universität Münster, CiMIC, CeNTech Heisenbergstraße 11 48149 Münster Germany cstra_01@uni-muenster.de; c Department of Chemistry and Materials Science, Aalto University FI-00076 Aalto Finland; d Faculty of Mathematics and Natural Sciences, Department of Chemistry and Biochemistry, Institute for Inorganic Chemistry, University of Cologne Greinstrasse 6 D-50939 Cologne Germany axel.klein@uni-koeln.de

## Abstract

The association of platinum(ii)-based luminophores, which is caused by metal⋯metal and π–π stacking interactions, has been actively exploited in supramolecular construction of photofunctional molecular materials. Herein, we describe a series of bimetallic complexes [{Pt(C^N^^/^*N)}_2_(CN)][BAr_4_^F^], containing cyanido-bridged cyclometalated Pt(ii) chromophore fragments (HC^N^N = 6-phenyl-2,2′-bipyridine, (benzyltriazolyl)-phenylpyridine, and pyrazolyl-phenylpyridine; HC^N*N = *N*-pentyl-6-phenyl-*N*-(pyridin-2-yl)pyridin-2-amine; ^/* denote five/six-membered metallocycles). These compounds are intensely phosphorescent at room temperature showing quantum yields up to 0.73 in solution and 0.62 in the solid state, which are generally higher than those of the mononuclear relatives [Pt(C^N^^/^*N)(CN)]. The complex cations bearing sterically unhindered ^−^C^N^N ligands readily assemble in solution, reaching the tetrameric species [{Pt(C^N^N)}_2_(CN)]_4_^4+^ as suggested by diffusion NMR spectroscopy. The size of the aggregates can be regulated by the concentration, temperature, and polarity of the solvent that allows to alter the emission from green to near-IR. In the solid state, the maximum of low-energy luminescence is shifted up to 912 nm. The results show that photophysical properties of discrete complexes and the intermolecular aggregation can be substantially enhanced by utilizing the rigid bimetallic units giving rise to novel dynamic light emitting Pt(ii) systems.

## Abstract 2

Linking two cyclometalated Pt(ii) fragments *via* cyanido bridge improves the luminescence and dramatically enhances the aggregation ability resulting in distinct sensitivity to the properties of the surrounding medium.

## Graphical abstract

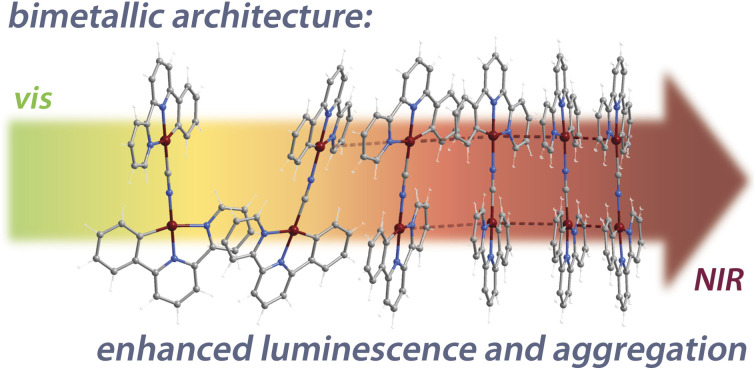

## Introduction

The rich and tunable photophysical properties of square planar Pt(ii) complexes are determined to a large extent by their prominent ability to participate in metal⋯metal and π–π stacking interactions, which make these compounds with d^8^ configuration an outstanding class of chromophores among other metal-based photofunctional materials.^1^ Metallophilic Pt⋯Pt interactions, assigned to the energetically favorable (up to 40 kcal mol^−1^)^1*e*^ overlap of 5d_*z*^2^_ and 6p_*z*_ orbitals from two adjacent metal centers, are manifested by a drastic red-shift of both the absorption and emission bands. This peculiar optical behavior, which is associated with the formation of low-energy metal-to-metal-to-ligand charge transfer states (^3^MMLCT), is often observed in solution and in the solid and has been applied in the design of organic light-emitting diodes (OLEDs),^2^ molecular sensing and recognition systems,^3^ bioimaging probes,^4^ and diverse responsive molecular materials.^5^

In particular, luminescent Pt(ii)-based pincer compounds bearing aromatic ligands have been extensively investigated by several groups in the view of their tendency for metallophilicity-driven aggregation.^1*b*–*d*,6^ The planar conjugated systems of such complexes facilitate efficient π–π stacking, the interplay of which with metal–metal and other types of non-covalent bonding defines their aggregation and supramolecular arrangement. Furthermore, large opportunities for modification of tridentate heterocyclic ligands significantly expand the ways to tune the physical and chemical functionalities of the assemblies. Due to the relative weakness of the aforementioned intermolecular interactions, the solid-state organization and the optical characteristics of these compounds often show sensitivity to external perturbation, resulting in distinct thermo-, vapo-^3*c*,7^ and mechanochromism^7*a*,8^ with potential use in chemosensing, gas/volatiles monitoring, and memory devices.

Introducing properly designed spacers between the planar tridentate pincer ligands and/or the ancillary ligands L/X of [Pt(Rterpy)L/X]^*n*+^ building blocks (Rterpy = derivatives of 2,2′;6′,2′′-terpyridine) provides a facile route to fabricate an impressive selection of supramolecular architectures and host–guest systems.^1*d*,3*d*,9^ On the other hand, tailoring specific substituents to the constituting ligands allows for regulating the bulkiness and the amphiphilic character of related Pt(ii) complexes [Pt(N^N^N)L/X]^*n*+^.^1*d*,3*d*,9^ Thus, weakly emissive ditriazolyl pyridine complexes bearing hydrophilic ethylene glycol pyridine ligands assemble into aggregates with strong photo- and electrochemiluminescence,^10^ and were further utilized to engineer protein-covered highly phosphorescent virus-like particles.^11^ Dinuclear compounds with hydrophobic tridentate terpyridine/diimidazolylpyridine ligands and oligomeric alkynyl spacers have been reported to form luminescent helix architectures, nanotubes and metallogels.^12^ Variation of the charge in substituents on diimidazolylpyridine ligands has been used for the construction of double salts affording infinite chains and nanofibers,^13^ while chiral helical ribbons as single component assemblies were obtained from complexes of alanine-functionalized terpyridine.^14^ In addition to the stereochemistry of the ligand environment and its hydro-/lipophilic properties, subtle intermolecular interactions between Pt(ii) chromophores with planar heteroaromatic ligands can be guided by the surrounding solvents and the counter ions, which influence the morphology of the aggregates.^15^ Successful manipulation of these factors together with molecular design is illustrated by the production of honeycomb network structures^16^ and luminescent pH-responsive porous polymers based on Pt(ii)-complex chromophores.^17^

No less interesting can be the effects of solvent polarity, hydrophobicity, and the constituting ions on the association, dynamics and the corresponding optical response of the Pt(ii) photofunctional species in fluid medium.^1*a*,3*d*,18^ For instance, the assembly of cyclometalated [Pt(C^N)(bpy)]^+^ (bpy = 2,2′-bipyridine) luminophores, drastically facilitated by halide ions, was employed for their detection in aqueous media and in biological objects.^19^ Changing the counter ions from bulky borate to chloride and the solvent from CHCl_3_ to hexane modulates the concentration-dependent emission from blue (monomer) to yellow (dimer) and orange (aggregate) for [Pt(N^C^N)(CNR)]^+^ complexes (HN^C^N = derivative of 2,6-dipyridyl-benzene).^20^ Ultimately, dinuclear terpyridine alkynylplatinum foldamers and tweezers undergo reversible intramolecular structural transformations and switch of host–guest interactions driven by the solvent, acidity and temperature dependent π–π stacking and Pt⋯Pt interactions, accompanied by distinct changes in the absorption and emission spectra.^5*g*,18*b*,21^

Most of the reports on supramolecular association of Pt(ii) luminophores correspond to mononuclear compounds. The relevant investigations of bi- and trimetallic complexes serving as building blocks are considerably less common and utilize almost exclusively terpyridine motifs [Pt(N^N^N)]^2+^,^12*b*,16*b*,17,21*b*,22^ albeit the presence of two or more metal fragments in one molecular entity could be beneficial for intermolecular interactions by increasing connectivity and dimensionality.

In our previous work, we have studied the cyclometalated cyanido Pt(ii) complexes [Pt(C^N^N)(CN)] with HC^N^N = 6-phenyl-2,2′-bipyridine (Hphbpy),^23^ (benzyltriazolyl)-phenylpyridine (Hphpytabn), and pyrazolyl-phenylpyridine (Hphpypz)^24^ alongside with the [Pt(C^N*N)(CN)] complex with HC^N*N = *N*-(2-phenylpyridine)-*N*-propyl-thiazole-2-amine^25^ and applied some of these components in the fabrication of halogen-bonded adducts and heterometallic coordination compounds *via* the ambidentate CN ligand,^24,26^ which has a well-documented tendency for bridging coordination.^27^ Prompted by a surprisingly limited number of cyanido-bridged cyclometalated Pt(ii) luminophores,^28^ we have chosen [Pt(C^N^N)]^+^ or [Pt(C^N*N)]^+^ (^ and * denote five and six-membered metallocycles, respectively^29^) fragments for the synthesis of bimetallic CN-bridged compounds. These complexes show intense phosphorescence in the solid state and in solution. In fluid medium, the aggregation is primarily governed by the organic ligand but also depends on the concentration, temperature, and solvent polarity.

## Results and discussion

### Synthesis and structural characterization

**Scheme 1:**
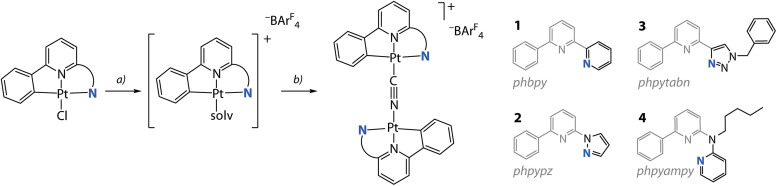
Synthesis of diplatinum cyanido complexes 1 to 4 from the chlorido precursors: (a) Na[BAr_4_^F^] in DMSO/CH_2_Cl_2_/water, 298 K, overnight; (b) NaCN in MeCN/MeOH, 298 K, overnight (74 to 91%).

The synthetic route to bimetallic compounds [{Pt(C^N^^/^*N)}_2_(CN)][BAr_4_^F^] (^−^BAr_4_^F^ = tetrakis[3,5-bis(trifluoromethyl)phenyl]borate) adapts the protocol for mononuclear cyanido derivatives [Pt(C^N^^/^*N)(CN)],^24^ see Scheme 1 and the ESI† for experimental details. We treated the [Pt(C^N^^/^*N)Cl] precursors (HC^N^N = Hphbpy, Hphpypz, Hphpytabn;^30^ HC^N*N = *N*-pentyl-6-phenyl-*N*-(pyridin-2-yl)pyridin-2-amine (Hphpyampy)^31^) with stoichiometric amounts of Na[BAr_4_^F^], thus removing the chlorido ligand to obtain highly soluble and labile [Pt(C^N^^/^*N)(solv)][BAr_4_^F^] intermediates (solv = dimethylsulfoxide (DMSO) or acetonitrile (MeCN)). The subsequent reaction with 0.5 eq. NaCN produced the salts [{Pt(C^N^^/^*N)}_2_(CN)][BAr_4_^F^] (HC^N^N = Hphbpy 1; Hphpypz 2; Hphpytabn 3; HC^N*N = Hphpyampy 4) in good yields (74 to 91%) as crystalline solids. The choice of the [BAr_4_^F^]^−^ counter ion is motivated by our observation of the poor solubility of these species with smaller anions (triflate, PF_6_^−^, BF_4_^−^). Compounds 1 to 4 are well-soluble in polar organic solvents (MeOH, acetone, MeCN, *N*,*N*-dimethylformamide (DMF)), while in CH_2_Cl_2_ and CHCl_3_ the solubility of 1 and 2 is low.

**Fig. 1:**
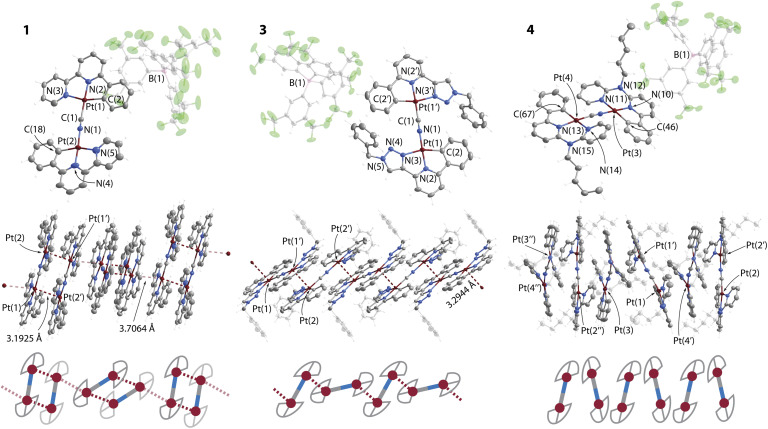
Molecular views (top) of the salts 1, 3^.^CH_2_Cl_2_ and 4 showing one of two (3^.^CH_2_Cl_2_) or three (4) independent cations found in the corresponding unit cells. The central and bottom views depict the columnar stacks formed by the cations. Displacement ellipsoids at 50% probability level, co-crystallized CH_2_Cl_2_ in 3^.^CH_2_Cl_2_ omitted for clarity.

The structures of complexes 1, 3·CH_2_Cl_2_ and 4 were determined by single crystal X-ray diffractometry, crystal data and refinement details are listed in Table S1 (ESI),† views of molecular ions and fragments of crystal packing are depicted in Fig. 1, S1 and S2 (ESI).† The compounds contain nearly planar bimetallic cations [{Pt(C^N^^/^*N)}_2_(μ-CN)]^+^, in which the [Pt(C^N^^/^*N)]^+^ fragments lie in the same plane and are linked *via* the μ-C,N–CN bridge. Recently, a conceptually similar series of complexes with diplatinum cyanido-bridged anions [NBu_4_][{Pt(C^N)(*p*-MeC_6_H_4_)}(μ-CN)] have been reported, which bear bidentate C^N cyclometalated motifs.^28^ The Pt centers are found in distorted square-planar environments, the structural parameters around the metal ions (Table S2, ESI†) are similar to those of the neutral monomeric congeners [Pt(C^N^^/^*N)(CN)],^24^ and of related complexes bearing C^N^^/^*N ligands.^3*c*,25,30*c*,32^

Complexes 1 and 2 reveal both crystallographic and chemical disorder in the packing of the cations. This prevents high quality refinement, particularly for 2, the structure of which is given in the ESI† only and is not discussed in detail. Nevertheless, the structures confirm the supramolecular arrangement of 1 and 2 in the solid state. Fig. 1 shows one of two disordered components, which were refined with occupancies of 0.49/0.51. Cations of 1 form dimers *via* metal–metal contacts (M⋯M) with distances of 3.193(2) Å for Pt(1)⋯Pt(2) and 3.417(3) Å for Pt(1A)⋯Pt(2A). Other Pt⋯Pt interactions between the disordered components Pt(1)⋯Pt(1A) and Pt(2)⋯Pt(2A) of 3.431(2) and 3.249(2) Å (averaged to 3.34 Å) might also contribute to the structure as they are shorter than the sum of two van der Waals radii (3.5 Å).^33^ The tetranuclear units adopt an eclipsed conformation of [{Pt(C^N^^/^*N)}_2_(CN)]^+^ motifs (torsion angle N–Pt(1)–Pt(2)–C ≈ 3°). In each component, two equivalent metal–metal interactions are nearly perpendicular to the planes of the bimetallic cations that provides a direct overlap of metal orbitals and rare face-to-face (or face-centered) π–π stacking^34^ of the metalated phbpy ligands (Fig. 1). A similar packing mode is found for cations of compound 2 (Fig. S1, ESI†). This geometry is unusual for Pt(ii) and Pd(ii) complexes with cyclometalated multidentate heteroaromatic ligands, in which unsupported metallophilic interactions typically form head-to-tail dimers, larger arrays or even more or less linear (M⋯M)_*n*_ chains of significantly staggered molecules.^3*c*,24,35^ A relatively close case of a tetraplatinum aggregate was described for a bimetallic isocyanide complex constructed from a carbazole-bridged biscyclometalating ligand (torsion angle C–Pt–Pt–C ≈ 25°).^36^ The π–π stacking interactions and longer Pt⋯Pt separations (averaged to 3.48 Å for two disordered components in 1) between orthogonally twisted tetraplatinum blocks further afford infinite aggregates in the dark brown and dark red crystals of 1 and 2, respectively.

The packing in the bright yellow 3·CH_2_Cl_2_ shows a ladder-type of polymeric assembly composed of [{Pt(C^N^N)}_2_(CN)]^+^ ions (Fig. 1). Each metal center is involved in one short intermolecular Pt⋯Pt contact (3.2944(4) Å) between adjacent {Pt(C^N^N)}^+^ motifs, which are oriented in a staggered fashion and with a torsion angle N–Pt(1)–Pt(2)–C of 113.7(1)°.

In compound 4, the presence of 6-membered metallocycles causes smaller geometric strain within the C^N*N group that apparently results in a less distorted square planar geometry with C(2)–Pt(1)–N(3) angles of about 168° in 4 compared with about 160^o^ in 1 and 3^.^CH_2_Cl_2_. Similarly to other Pt(ii) compounds with congener ligands having large bite angles,^37^ the amino-bipyridine moiety in 4 is non-planar. This structural feature enhanced by steric repulsion of the C–H groups of the CN-bridged fragments, prevents efficient π–π stacking and metal–metal interactions, *i.e.* allows considering crystals of 4 as containing discrete molecular ions.

### Solid-state luminescence

**Fig. 2:**
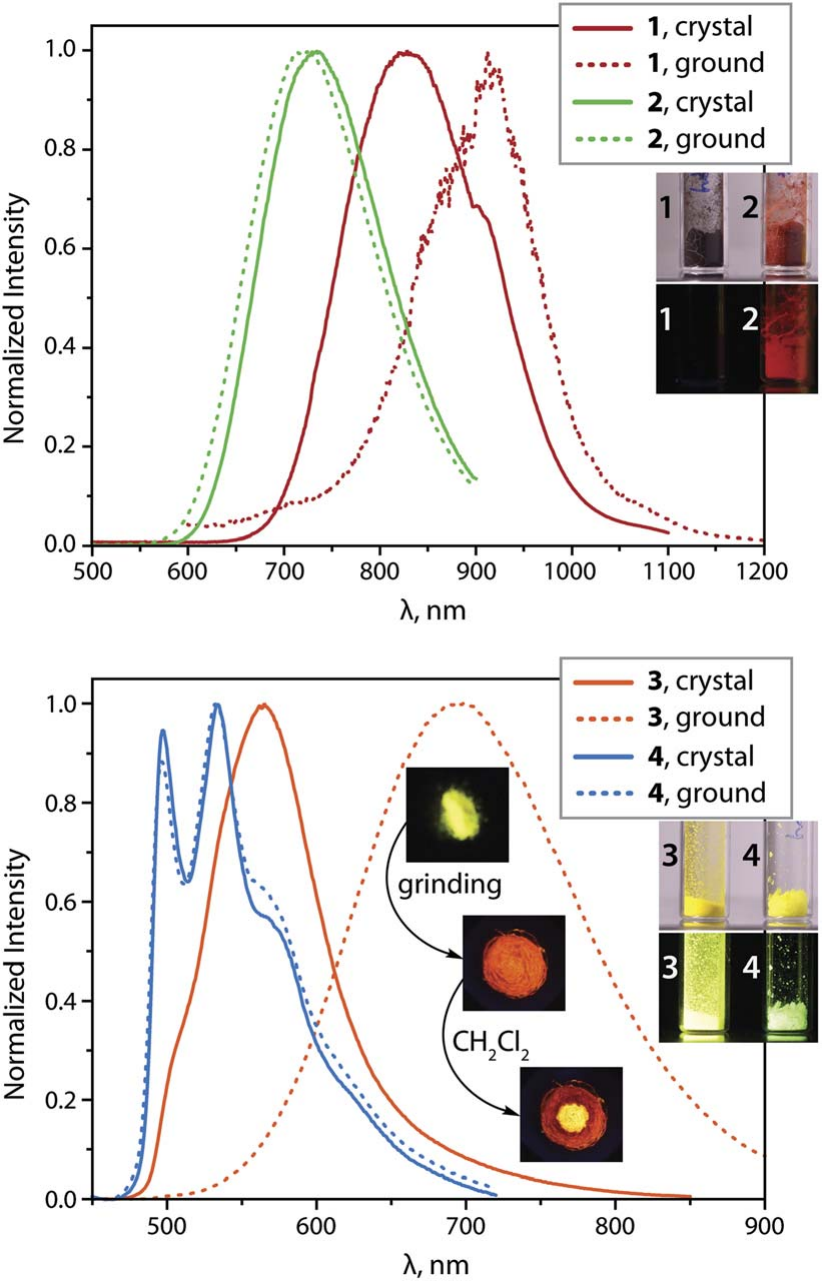
Normalized photoluminescence spectra of complexes 1 to 4 in the solid state at 298 K (*λ*_exc_ = 375 nm; the inset photos show the appearance of crystalline samples under ambient and 365 nm UV light, and the effect of grinding and solvent on the emission of 3 (vacuum dried crystallization solvent-free); the kink in the spectrum of 1 at 900 nm is an artifact due to the change of the grid in the monochromator).

The pertinent photophysical properties for complexes 1 to 4 are listed in Table 1, the emission spectra at 298 K and at 77 K are shown in Fig. 2, S4 and S5, ESI.† The photoluminescence (PL) characteristics of crystalline samples generally reflects the packing and intermolecular interactions of the diplatinum cations. Compound 1 with extensive metallophilic interactions is the lowest energy emitter showing a structureless band peaking at 828 nm and a quantum yield below 0.04 (we were not able to determine accurately absolute quantum yields due to the detector of the integrating sphere being limited to *λ*_em_ = 750 nm). A relatively short lifetime of 65 ns evidently arises from large non-radiative rates (1.54 × 10^7^ s^−1^ > *k*_nr_ > 1.48 × 10^7^ s^−1^), which is not exceptional for near-IR luminescence. The excited state probably corresponds to a metal-to-metal-to-ligand charge-transfer configuration with triplet multiplicity (^3^MMLCT or ^3^[dσ* → π*] where the dσ* orbital corresponds to the metal–metal interaction). These are typical for Pt(ii) complexes aggregated *via* Pt⋯Pt contacts and showing long-wavelength phosphorescence with *λ*_em_ up to about 1000 nm.^1*c*,1*f*,2*c*,2*e*,38^ In comparison with the red emissive mononuclear congener [Pt(phbpy)(CN)] (*λ*_em_ = 710 nm),^24^ which crystallized as Pt⋯Pt dimers, assembled further *via* head-to-tail π–π stacking, 1 presents a significant bathochromic shift.

**Table 1:** Photophysical properties of complexes 1 to 4 in the solid state and in solution

	State	*λ*_em_, nm		*τ*_av_,^a^ μs		*Φ*_L_ (±2%/±5%)		b *k*_r_, 10^5^ s^−1^		b *k*_nr_, 10^5^ s^−1^	
		298 K	77 K	c298 K	77 K	c298 K	77 K	c298 K	77 K	c298 K	77 K
1	Crystal	828	800	0.065 ± 0.001	0.133 ± 0.002	<0.04^d^	<0.04^d^	<6.2	<3.1	>148 (<154)^e^	>72 (<75)^e^
	Ground	912	∼790	0.089 ± 0.001	0.362 ± 0.009	<0.04	<0.04^d^	<4.5	<1.1	>108 (<112)^e^	>27 (<28)^e^
	CH_2_Cl_2_	546, 575sh	—	3.33 ± 0.02	—	0.25	—	0.75 ± 0.06	—	2.25 ± 0.08	—
	2-MeTHF	—	516, 556, 595sh	—	9.95 ± 0.02	—	0.95^f^		0.95 ± 0.05		<0.1
2	Crystal	734	808	0.548 ± 0.005	0.87 ± 0.02	0.26^d^	0.16^d^	4.7 ± 0.4	1.8 ± 0.6	13.5 ± 0.6	9.7 ± 0.8
	Ground	724	771	0.604 ± 0.006	1.20 ± 0.04	0.27^d^	0.22^d^	4.5 ± 0.4	1.8 ± 0.5	12.1 ± 0.5	6.4 ± 0.8
	CH_2_Cl_2_	499, 533, 571sh	600, 705^f^	14.39 ± 0.03	3.125 ± 0.009 (600 nm)	0.72	0.76^f^	0.50 ± 0.01	—	0.20 ± 0.02	—
	2-MeTHF	—	490, 528, 564	—	15.11 ± 0.04	—	0.95		0.63 ± 0.03		<0.06
3^g^	Crystal	565	575	0.63 ± 0.01	2.72 ± 0.02	0.62	0.92	9.8 ± 0.5	3.4 ± 0.2	6.0 ± 0.8	0.3 ± 0.2
	Ground	698	653	1.208 ± 0.004	2.67 ± 0.02	0.48	0.54	4.0 ± 0.2	2.0 ± 0.2	4.3 ± 0.2	1.7 ± 0.2
	CH_2_Cl_2_	503, 538, 575sh	620^f^	13.14 ± 0.02	3.524 ± 0.003	0.73	0.95	0.56 ± 0.02	2.7 ± 0.1	0.20 ± 0.02	<0.28
	2-MeTHF	—	495, 530	—	11.32 ± 0.01 (495 nm)	—	0.95		0.84 ± 0.05		<0.1
			570, 620		4.25 ± 0.02 (620 nm)						
4	Crystal	534	496	14.0 ± 0.2	14.5 ± 0.4	0.50	0.64	0.36 ± 0.02	0.44 ± 0.05	0.36 ± 0.03	0.25 ± 0.07
	Ground	532	501	6.2 ± 0.3	19.7 ± 0.2	0.24	0.71	0.38 ± 0.05	0.36 ± 0.03	1.2 ± 0.1	0.15 ± 0.03
	CH_2_Cl_2_	493, 529, 562sh	487, 526, 560sh^f^	2.66 ± 0.02	38.3 ± 0.1	0.07	0.95	0.26 ± 0.08	0.25 ± 0.01	3.5 ± 0.1	<0.02
	2-MeTHF	—	488, 526, 563sh	—	38.4 ± 0.1	—	0.95		0.25 ± 0.01		<0.02

a Amplitude-weighted average lifetimes determined by the equation *τ*_av_ = ΣA_*i*_*τ*_*i*_, A_*i*_ = weight of the *i*-th component, *λ*_exc_ = 375 nm.

b *k*_r_ and *k*_nr_ were estimated as *Φ*_L_/*τ* and (1 − *Φ*_L_)/*τ*, respectively.

c Under an inert atmosphere of Ar.

d The detector of the integrating sphere is limited to *λ*_em_ = 750 nm.

e Upper limit calculated for *Φ*_L_ = 0.

f Diluted solution CH_2_Cl_2_ : MeOH 1 : 1 v/v.

g Vacuum dried solvent-free crystals of 3·CH_2_Cl_2_.

Grinding the crystals of 1 red shifts the 298 K emission to 912 nm with little effect on the intensity and decay rates. According to powder XRD (PXRD) measurements (Fig. S3, ESI†), ground sample of 1 shows substantial loss of crystallinity that likely perturbs intermolecular interactions. A decrease in the emission energy could result from more accessible structural relaxation (*i.e.*, stabilization) of the triplet excited state in a less rigid amorphous phase. At 77 K, both crystalline and the ground samples exhibit similar luminescence profiles with maxima at about 800 nm (Fig. S4, ESI†) and virtually the same quantum efficiencies as those at ambient conditions.

The deep red luminescence of microcrystalline sample 2 (*λ*_em_ = 734 nm) correlates with the presence of short metal⋯metal contacts, as suggested by the XRD analysis. This is also supported by the photophysical characteristics of the related solid species [Pt(phpypz)(CN)] (*λ*_em_ = 595 nm, *Φ*_L_ = 0.17),^24^ and [Pt(phpypz)Cl] (*λ*_em_ = 534 nm),^30*c*^ which reveal only intermolecular π–π stacking interactions. The quantum yield for 2 (*Φ*_L_ = 0.26) reaches a relatively high value among the deep red Pt(ii) emitters,^2*c*,35*d*^ conceivably due to suppression of non-radiative decay with respect to 1 (Table 1). Mechanical force causes only slight perturbation of luminescence of 2 at 298 K, which implies stability of molecular packing, as confirmed by minor changes in PXRD pattern (Fig. S3, ESI†). Cooling to 77 K shifts the emission wavelength to 808 nm (771 nm for the ground sample) that might be caused by contraction of Pt⋯Pt contacts occurring at low temperature.^35*c*^

The crystalline dimer 3 (vacuum dried crystallization solvent-free sample was used for the measurements) exhibits the most intense room-temperature emission (*λ*_em_ = 565 nm, *Φ*_L_ = 0.62) within the studied series. Although removal of co-crystallized solvent (CH_2_Cl_2_) through grinding affects the PXRD pattern for 3 (Fig. S3, ESI†), the dried material clearly retains its crystalline nature. The luminescence of 3·CH_2_Cl_2_, measured for crystals preserved in mother liquor (*λ*_em_ = 575 nm) is rather similar to that of dried crystals (Fig. S5, ESI†). This suggests that intermolecular interactions between the chromophore fragments in 3 resemble those in 3·CH_2_Cl_2_. The featureless band in the yellow region of the spectrum can be ascribed to Pt⋯Pt and π–π contacts, found in the solid state (Fig. 1). Noteworthy, the quantum efficiency of 3 is substantially higher than that of the complexes [Pt(phpytabn)(CN)] (*λ*_em_ = 535 nm, *Φ*_L_ = 0.07, *k*_r_ = 3 × 10^4^ s^−1^)^24^ and [Pt(phpytabn)Cl] (*λ*_em_ = 500 nm, *Φ*_L_ = 0.15, *k*_r_ = 6 × 10^4^ s^−1^)^30*b*^ bearing the same metalated ligand. The latter compounds showed vibronic progressions indicating a large contribution of intraligand (IL = π–π*) character describing the T_1_ excited state. The boost in *Φ*_L_ of 3 then mainly stems from the increase of radiative rate (*k*_r_ = 9.8 × 10^5^ s^−1^) that is tentatively assigned to primarily ^3^MMLCT origin of the lowest lying triplet state, where the charge transfer character and metal⋯metal interactions enhance spin–orbit coupling and accelerate the radiative relaxation. Upon grinding, yellow phosphorescence of 3 changes to red (*λ*_em_ = 698 nm, *Φ*_L_ = 0.48) but retains good quantum yields because of a rather low rate of radiationless decay. The PXRD pattern for ground 3 indicates an amorphous character. The phase transition is reversible and yellow emission is restored by treating the ground material with a droplet of CH_2_Cl_2_, alcohols or acetone (Fig. 2 and S5, ESI†). Mechanically-induced bathochromic shift of luminescence has been documented for pincer and other Pt(ii) compounds and can be attributed to strengthening of the metal⋯metal and π–π interactions.^5*l*,5*n*,7*a*,39^ A similar effect, *i.e.* the shortening of Pt⋯Pt contacts in 3 upon grinding due to removal of structural constraints is also suggested from the deep-orange color of the ground sample *vs.* the yellow neat crystals. Analogously to 1, we assume that grinding of 3 leads to an energetically lower-lying T_1_ state, further contributing to distinct mechanoluminochromism. This hypothesis is in line with the blue-shift of the emission of both amorphous 1 (122 nm) and 3 (45 nm) at 77 K (Table 1, Fig. 2 and S4, ESI†), where molecular motion and structural changes in the excited state are noticeably restricted.

Alternatively, aggregates with extended metal–metal connectivity (*e.g.* trimer species with Pt⋯Pt⋯Pt chains) could account for low-energy emission of amorphous 3. This would require substantial intermolecular motion with respect to packing in the parent crystal and is probably not favorable due to steric hindrance imposed by out of plane benzyl substituents. Moreover, the absence of trimeric species of 3 in frozen solution (see below) also presumes their unlike formation in amorphous solid.

In contrast to compounds 1, 2 and 3 with charge-transfer-related phosphorescence, the vibrationally structured emission band of crystalline complex 4 (*λ*_em_ = 534 nm, *Φ*_L_ = 0.50) is principally assigned to the ^3^IL character of the excited state localized on the organic fragment. The radiative rate of 4 (*k*_r_ = 3.6 × 10^4^ s^−1^) is substantially lower than those for other compounds studied herein, which also supports the given assignment. The optical properties of solid 4 (*λ*_em_, *Φ*_L_, *k*_r_) are weakly affected by grinding or cooling. This behavior correlates well with the lack of intermolecular metal⋯metal and π–π stacking interactions due to the non-planarity and bulkiness of the metalated ligand mentioned above (Fig. 1). In comparison with the aforementioned congener complexes [NBu_4_][{Pt(C^N)(*p*-MeC_6_H_4_)}(μ-CN)]^28^ (showing emission from states with IL character and quantum yields ranging from 3 to 20% in the solid state), compound 4 constitutes a substantially more efficient luminophore due to a much slower *k*_nr_ despite a similar nature of the excited state.

### MS and NMR studies in solution

Mass spectroscopic (MS) data of complexes 1 to 4 confirm the presence of bimetallic cations in solution. The ESI-MS(+) display dominating signals of singly charged molecular species [{Pt(C^N^^/^*N)}_2_(CN)]^+^, the experimental *m*/*z* values and the isotopic patterns completely agree with calculated patterns (Fig. S6, ESI†).

The ^1^H nuclear magnetic resonance (NMR) spectra of 1 to 4 recorded in dilute solutions (*c* ≈ 1 mM) demonstrate two partially overlapping sets of resonances, which correspond to two inequivalent parts {Pt(C^N^^/^*N)}^+^ (Fig. S7 and S8, ESI†) caused by the non-symmetric CN^−^ bridge. In addition, the spectra show the signals of the ^−^BAr_4_^F^ counter ions (*δ* = 7.7 to 7.8 ppm and a singlet at 7.5 to 7.7 ppm), the ratio of integral intensities confirm the proposed stoichiometry [{Pt(C^N^^/^*N)}_2_(CN)][BAr_4_^F^]. Each of the complexes shows two signals in the ^195^Pt NMR spectrum (*c* ≈ 10 mM) confirming the presence of chemically non-equivalent metal centers. The values of chemical shifts (−3614 to −3457 ppm for low field and −4019 to −3852 ppm for high field signals) are found in the range, previously reported for Pt(ii) complexes with cyclometalated ligands.^40^

**Fig. 3:**
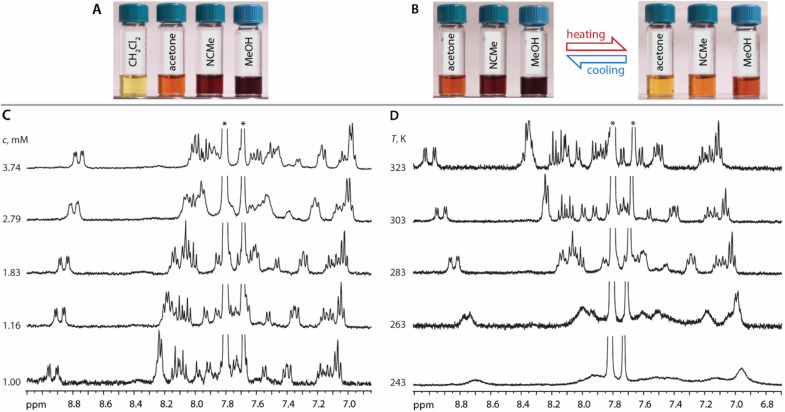
(A) Photographs of solutions of 1 in CH_2_Cl_2_, acetone, MeOH, MeCN (*c* = 0.7 mM, 298 K). (B) *T*-dependent reversible color changes of solutions of 1 in acetone, MeOH, MeCN (*c* = 0.7 mM). (C) Variable concentration 400 MHz ^1^H NMR spectra of 1 (acetone-*d*_6_, 298 K). (D) Variable *T* 400 MHz ^1^H NMR spectra of 1 (acetone-*d*_6_, *c* = 1.16 mM). Asterisks denote signals of ^−^BAr_4_^F^ counter ion.

In CD_2_Cl_2_, both 1 and 2 have low solubility (less than 1 mM) and show limited concentration dependence giving pale yellow solutions (Fig. 3A). Thus, in moderately polar chlorinated solvents, we assume the prevalence of discrete molecular ions.

In more polar solvents (acetone, MeOH, MeCN), even at relatively low concentrations (*e.g.*1 at *c* = 0.7 mM), the solutions of 1 and 2 are substantially darker, suggesting intermolecular association (Fig. 3A). For a detailed NMR analysis, we chose compound 1, which shows appreciable aggregation in acetone without significant raise of solution viscosity. The increase of concentration of 1 (1.00 → 3.74 mM) or lowering of the temperature (323 → 243 K, *c* = 1.16 mM) result in moderate high-field shift and broadening of the ^1^H signals accompanied by more intense coloring (Fig. 3). This behavior resembles that of mononuclear complexes of the types [Pt(N^N^N)(Me)]^+^,^41^ [Pt(N^N)(S^O)]^+^,^42^ [Pt(N^C^N)(MeCN)]^+^,^35*c*^ or [Pt(N^N)Cl_2_],^43^ and is related to the formation of aggregates.

Previously, concentration-dependent supramolecular assembly of Pt(ii) complex cations was analyzed by diffusion NMR spectroscopy,^35*c*,42^ which allows for measuring the diffusion coefficient (*D*) of the species existing in solution long enough in the NMR timescale.^44^ Employing the Stokes–Einstein equation *D* = *kT*/6π*ηr*_H_ (*k*: Boltzmann constant, *T*: absolute temperature, *η*: viscosity of the solvent, *r*_H_: hydrodynamic radius of the particle), it is possible to obtain a structural estimate (*r*_H(av)_) of the average size of the aggregates. The latter serves as semi-quantitative measure for the oblate cation 1 because the equation is applicable for spherical particles much larger than the solvent molecule. Nevertheless, the relative trend in the change of *r*_H(av)_ with concentration can provide valuable information concerning the aggregation number (*N*). *N*, *i.e.* the number of constituting fragments, is defined as the ratio of hydrodynamic volumes of the supramolecular particle (*V*_H_) and of the single block 
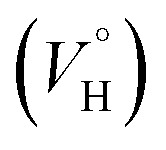
:^44^
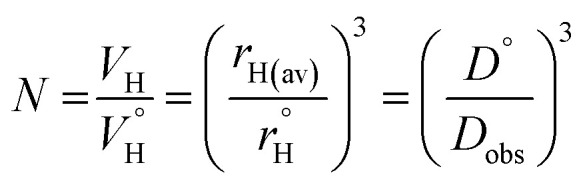


**Fig. 4:**
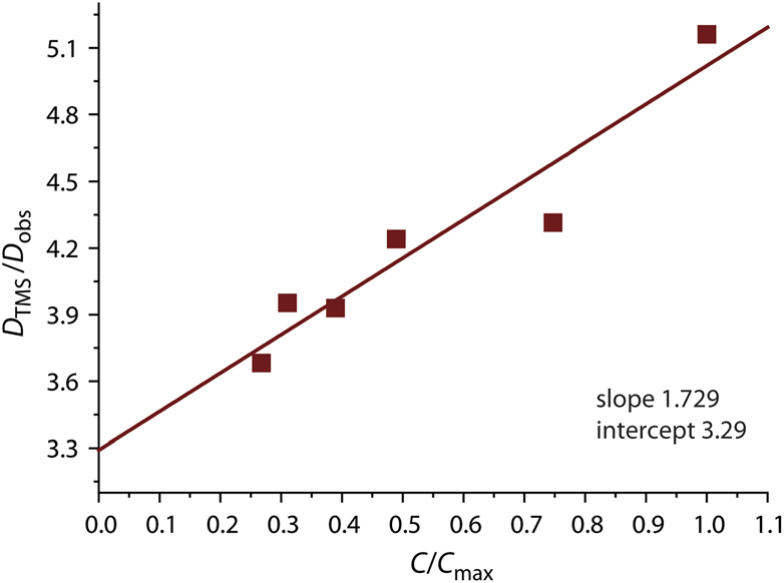
Concentration dependence of the *D*_TMS_/*D*_obs_ ratio for complex 1 (acetone-*d*_6_, 298 K).

The use of internal standard with negligible aggregation and therefore *r*_H_ = constant (tetramethylsilane, TMS) is a facile way to eliminate the effect of the solvent (*η*) and temperature. The *D*_TMS_/*D*_obs_ ratio then depends on the *r*_H(av)_ only and *D*° corresponds to infinite dilution (*c* = 0, Fig. 4). The diffusion coefficients determined from NMR measurements and the calculated average aggregation numbers are given in Table 2.

**Table 2:** Concentration dependences of diffusion coefficients (*D*) for TMS and 1, and average aggregation number (*N*) for^a^1

*c*[1], mM	*c*/*c*_max_	*D*_TMS_, 10^−9^ m^2^ s^−1^	*D*_obs_, 10^−9^ m^2^ s^−1^	*D*_TMS_/*D*_obs_	*N*
3.74	1.00	3.36	0.651	5.16	3.89
2.79	0.75	3.37	0.781	4.31	2.27
1.83	0.49	3.38	0.797	4.24	2.16
1.46	0.39	3.38	0.860	3.93	1.72
1.16	0.31	3.38	0.855	3.95	1.75
1.00	0.27	3.38	0.918	3.68	1.41

a From 400 MHz ^1^H NMR spectroscopy in acetone-*d*_6_ at 298 K.

At the concentration of 1 mM in acetone complex 1 shows a substantial degree of aggregation (*N* = 1.41) that also correlates with the visual observation of a brown solution (*c* = 1 mM, Fig. 3A). Increase in concentration causes fast growth of the average size of the aggregates. At *c* = 3.74 mM, *N* reaches the value of 3.89, *i.e.* an appreciable contribution of tetramer (octaplatinum) species [{Pt(phbpy)}_2_(CN)]_4_^4+^ might be expected.

### Photophysics in solution and theoretical analysis

**Fig. 5:**
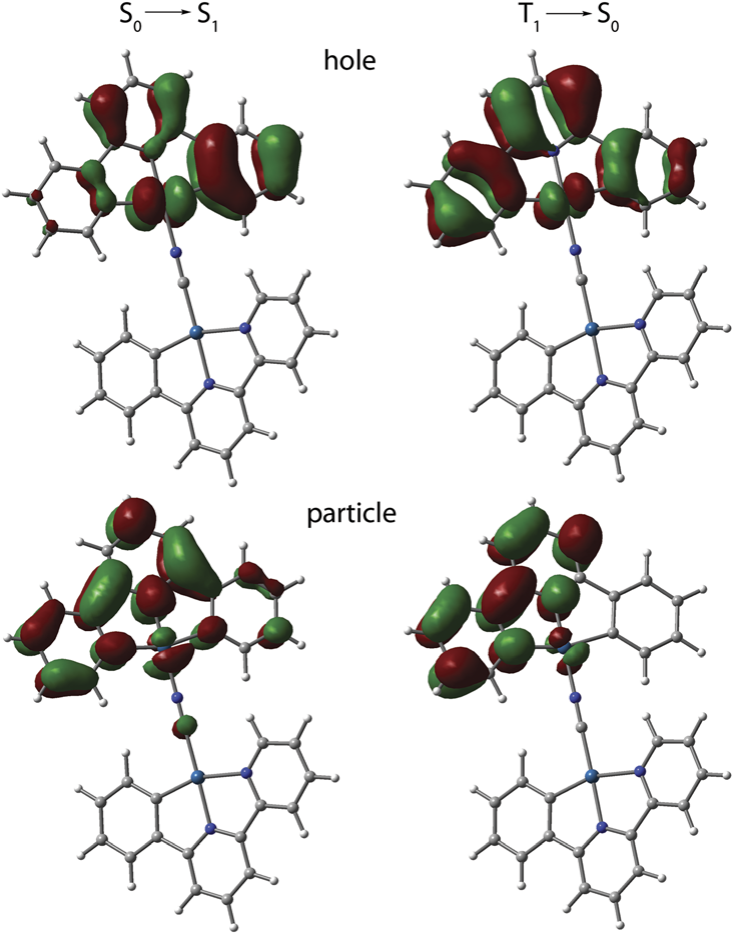
TD-DFT-calculated natural transition orbital hole-particle pairs for the vertical S_0_ → S_1_ and T_1_ → S_0_ transitions for cation of 1 in CH_2_Cl_2_ at the optimized excited state geometry.

The photophysical behavior of 1 to 4 in solution in the absence of aggregation is defined by the cyclometalated ligand and resemble those of the mononuclear congeners [Pt(C^N^^/^*N)(CN)] (1_mono to 4_mono).^23,24,31^ The UV-vis absorption spectra of 1 to 4 in CH_2_Cl_2_ (*c* < 0.1 mM, Fig. S9, data in Table S3, ESI†) display moderately intense bands in the region 300 to 380 nm, and weaker low-energy absorptions around 400 nm with tails extending to 440 to 450 nm. In all cases, the TD-DFT-predicted S_0_ → S_1_ bands (MN15 functional) are assigned to transitions into states with mixed MLCT/IL character, which are localized on one {Pt(C^N^^/^*N)} fragment (N_CN_ bound) with minor participation of cyanido orbitals (Fig. 5, S10, and Table S4, ESI†).

**Fig. 6:**
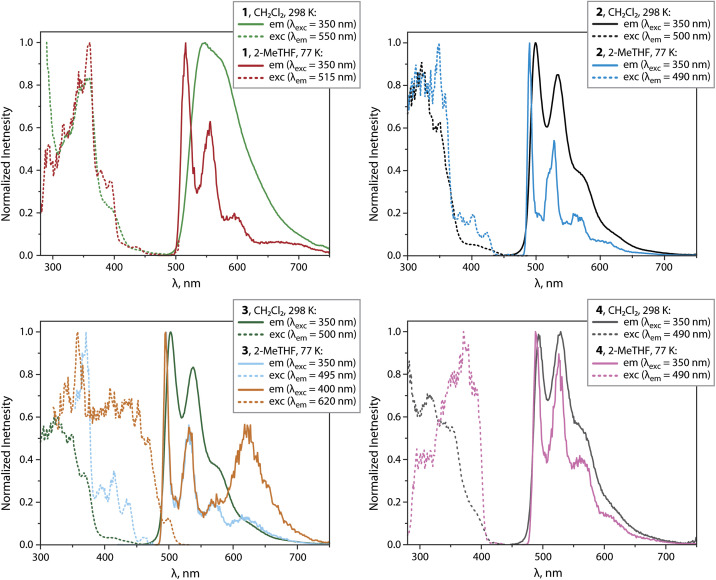
Normalized emission and excitation spectra of complexes 1 to 4 in CH_2_Cl_2_ (298 K) and frozen 2-MeTHF (77 K).

Compounds 1 to 4 show intense PL under oxygen-free conditions in CH_2_Cl_2_ solutions at 298 K. The PL spectra for 2 to 4 show bands in the range 500 to 700 nm with well-resolved vibrational progressions (Fig. 6 and Table 1). The maxima are found at very similar wavelengths (*λ*_em_ = 493 to 503 nm).

For 1, a broad yet almost unstructured band with a bathochromically shifted maximum at 546 nm is observed. Compounds 2 and 3 show the highest quantum yields reaching the value *Φ*_L_ = 0.73. With respect to neutral mononuclear analogues 1_mono to 4_mono (ref. 23, 24 and 31) (*Φ*_L_ = 0.10 to 0.49, Table S5 and Fig. S11, ESI†), 1 to 3 show improved emission efficiencies, while the *Φ*_L_ of 4 (0.07) is lower than that of 4_mono (0.33). The enhancement for the bimetallic compounds 1 to 3 arises from markedly lower non-radiative rates *k*_nr_, and moderately increased radiative rates *k*_r_ (Tables 1 and S5, ESI†). The latter vary from 0.26 × 10^5^ (4) to 0.75 × 10^5^ s^−1^ (1) and do not change significantly with temperature, indicating phosphorescence. At 77 K in 2-methyltetrahydrofuran (2-MeTHF) with *λ*_exc_ = 350 nm, all compounds show structured emission bands with maxima ranging from 488 to 516 nm, and quantum yields close to unity. For 1 and 3, low-energy bands were found at 675 and 625 nm, which probably correspond to a certain degree of aggregation.

Computational analysis confirms that the T_1_ → S_0_ emission for 1 to 4 involves only one {Pt(C^N^^/^*N)} motif (Fig. 5 and S12, ESI†). Similarly to the excitation, the emission corresponds to an intraligand-centered excited state (IL) perturbed by the admixture of MLCT character. The DFT-calculated vibrationally resolved spectra (Table S6†) reasonably correlate with the experimental data. For complex 1, the natural transition orbitals for the phosphorescent deactivation involve the entire C^N^N ligand and show a pronounced degree of MLCT character for the excited state, which is very likely responsible for the observed rather featureless emission pattern. For 2 to 4, the relaxation of the T_1_ state mostly takes place on the C^N (phenyl-pyridine) part of the metalated system, with very minor participation of one N-heterocycle (Fig. S12, ESI†) that account for a smaller CT character of this electronic state.

Since NMR experiments showed that aggregation of these complexes is favored by increasing solvent polarity, we studied the PL behavior of compounds 1 and 2 in polar solvents acetone, MeCN, and MeOH. In dilute solutions (O.D. <0.1), the emission spectra of these species in polar medium are identical to those in CH_2_Cl_2_ and originate from discrete cations. In acetone at *c* = 1 mM (*i.e.* at the average aggregation number *N* = 1.4, see Table 2), 1 exhibits a broad near-IR band at *λ*_em max_ = 844 nm together with higher energy residual signal peaking at 575 nm, Fig. S13, ESI,† which presumably emerge from the dimer (MMLCT excited state) and the monomer (mixed IL/MLCT excited state), respectively. At a concentration of 2 mM (*N* > 2.16) only the low-energy signal of the aggregate (dimer) is observed. Further increase of concentration or solvent polarity did not lead to other red-shifted bands of larger species probably due to negligible intensity of their emission.

The DFT-optimized geometry of the dimer [1]_2_^2+^ based on crystallographic data generally matches experimental structure (Fig. S14, ESI†) and features two diplatinum units linked through of Pt⋯Pt and π–π interactions. Because of the disorder found in the crystal, we considered two possible orientations of the cyclometalated ligands in the stacked molecules (NNC:CNN dimer A; NNC: NNC dimer B). Pt⋯Pt distances in the ground state vary from 3.382 to 3.426 Å. The lowest lying triplet excited state for these dimeric species (^3^MMLCT) is characterized by a strong contraction of one Pt⋯Pt distance (to *ca.* 2.8 Å). Calculated phosphorescence wavelengths strongly depend on the conformation (785 and 698 nm for dimer A and B, respectively). These values are relatively close to that observed at room temperature for 2 mM (*N* > 2.16) acetone solution (*λ*_em_ ≈ 840 nm Fig. S13, ESI†).

For comparison, we optimized dimeric and trimeric assemblies of 1 with more conventional head-to-tail orientation of the cyclometalated fragments (Fig. S14, ESI†). Noteworthy, head-to-tail geometry (dimer C) is energetically more favorable by 0.53 and 0.17 eV than face-to-face models (dimers A and B). The calculated ^3^MMLCT emission of the head-to-tail species [1]_2_^2+^ was hypsochromically shifted to 661 nm. Near-IR phosphorescence at *λ*_em_ = 782 nm was predicted for a freely optimized trimer, which is expected to prevail at higher concentration (*ca.* 3 mM, aggregation number *N* = Table 2). The face-to-face aggregates of 1 therefore might dominate the emission properties under ambient conditions in polar solvents.

**Fig. 7:**
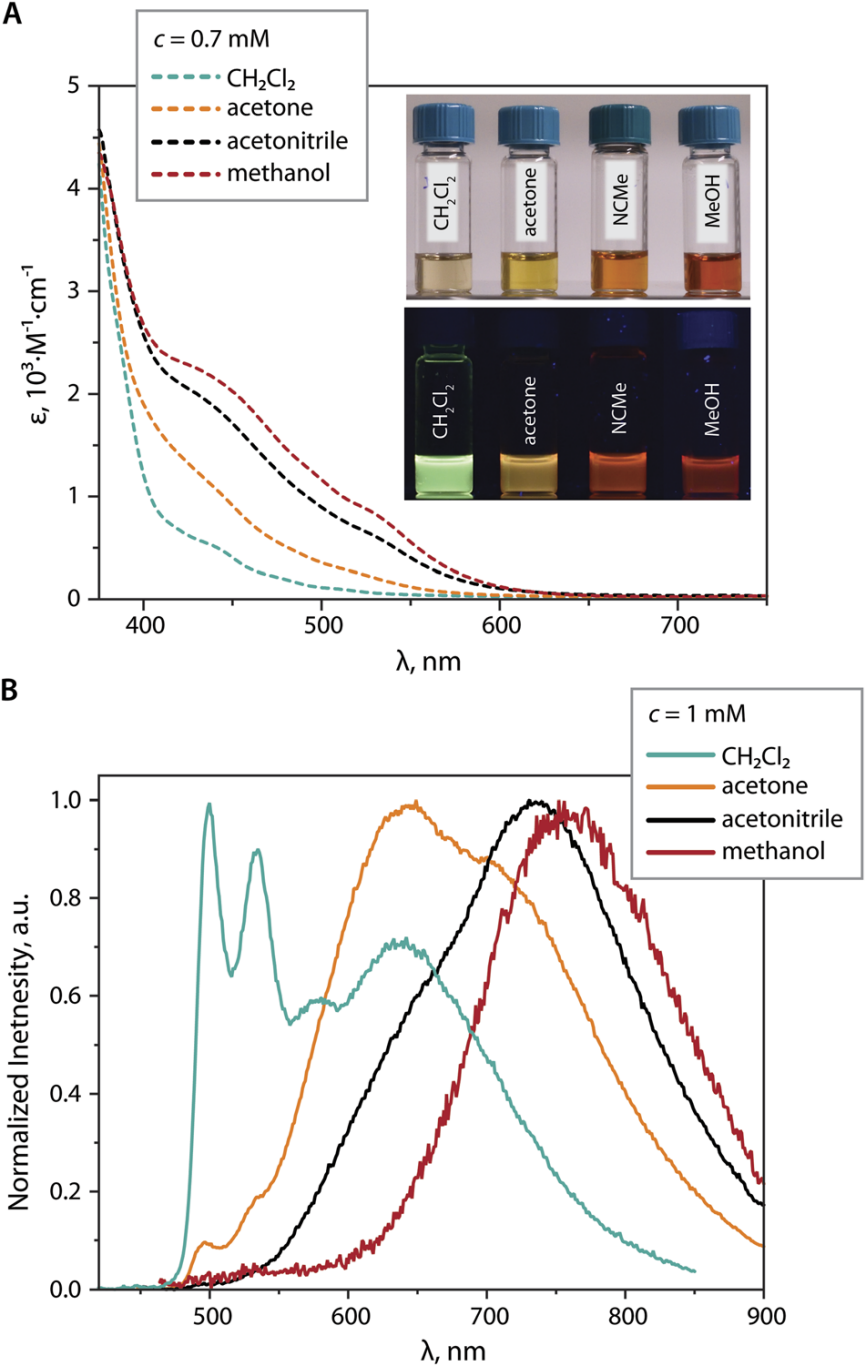
(A) UV-vis absorption spectra of 2 in CH_2_Cl_2_, acetone, MeCN, MeOH (*c* = 0.7 mM, 298 K; the photo shows the corresponding solutions under ambient, top, and UV (365 nm) light, bottom). (B) Normalized emission spectra of 2 in CH_2_Cl_2_, acetone, MeOH, and MeCN solutions (*c* = 1 mM, 298 K, *λ*_exc_ = 400 nm).

At a concentration of 1 mM in CH_2_Cl_2_, the PL spectrum of 2 (Fig. 7B) shows a main structured band peaking at 499 nm, which is assigned to the monomer, together with a relatively weak broad signal at 630 nm assigned to the contribution of the dimer and the excimer. The latter is identified by the rise component of the lifetime monitored at 640 nm (Fig. S15, ESI†).^45^ In the more polar solvent acetone,^46^ the intensity of the high-energy band (monomer) is dramatically lower, and the maximum of the broad band is shifted to 640 nm (presumably dimer and some excimer), along with the appearance of a shoulder at 715 nm (presumably trimer). Further increase in solvent polarity (MeCN) causes almost complete disappearance of the monomer emission, the decrease of the orange band (*i.e.* the amount of dimer), and the growth of a signal peaking at 735 nm (trimer). Ultimately, in MeOH the featureless far-red emission (*λ*_em_ = 755 nm) likely originates from the trimer or higher aggregates. More intense absorptivity of 0.7 mM MeCN and MeOH solutions of 2*vs.* that in CH_2_Cl_2_ in the visible region (Fig. 7A), together with the absence of the rise time in the emission decay curves, allows to rule out the formation of exciplexes, and to assign the low-energy emission band (*λ*_em_ > 700 nm) primarily the ground state associate [{Pt(phpypz)}_2_(CN)]_3_^3+^.

In contrast to [1]_2_^2+^, DFT-optimization of the ground state dimer [2]_2_^2+^ derived from XRD determined structure did not retain the face-to-face motif but results in the staggered configuration with substantial overlap between the cyclometalated ligands (dimer A, Fig. S16, ESI†) and predicted phosphorescence at 671 nm. On the other hand, head-to-tail aggregates [2]_2_^2+^ (dimer B, energetically lower than dimer A by 0.62 eV) and [2]_3_^3+^ were predicted to emit at 573 and 685 nm, respectively, which adequately correlates with behavior of 2 in different solvents (Fig. 7B).

The presence of several luminescent specimens of various geometries and resulting from stepwise assembly thus can occur in fluid medium for 1 and 2. This is also supported by the emission of 1 and 2 in optically dilute frozen glasses (CH_2_Cl_2_ : MeOH 1 : 1 v/v) at 77 K (Fig. S17, ESI†), where three distinct bands in each case can be assigned in the order of descending energy to a monomer, dimer, and trimer (or geometrically alternative dimer).

In the case of 3 measured in a CH_2_Cl_2_ : MeOH mixture at 77 K, the only band maximized at *ca.* 620 nm and the lack of NIR signals (Fig. S17, ESI†) indicate that aggregation produces dimer-like species. The stereochemistry of the DFT-simulated assembly [3]_2_^2+^ (Fig. S18, ESI†) correlates well with that in crystal, giving calculated phosphorescence from an ^3^MMLCT state at 584 nm (*cf.* 565 nm for crystalline 3, Table 1).

Comparison with the corresponding spectra of compounds 1_mono to 4_mono recorded under the same conditions (CH_2_Cl_2_ : MeOH 1 : 1 at 77 K, Fig. S11, ESI†) clearly shows the higher tendency of the cationic bimetallic complexes 1 and 2 towards aggregation and lower energy luminescence *vs.* their neutral monoplatinum congeners.

The assembly of planar Pt(ii) cations due to solvophobic interactions^5*g*,16*a*,18*a*,20,21*b*^ was described for a number of complexes. In particular, the examples not related to low solubility and formation of nanostructures include the red shift of both absorption and emission that occurs on changing MeOH to water for [Pt(C^N^N)(CNR)]Cl and [Pt(N^C^N)(CNR)]_2_(SO_4_) species, which oligomerize in aqueous medium.^18*a*,47^ It was shown recently that the host–guest complex of a bimetallic [Pt(N^N^N)]_2_-calix[4]arene molecular tweezer and [Pt(C^N^C)(CNR)] guest is solvent-dependent and is formed in MeOH but disassembled in CH_2_Cl_2_.^21*b*^ On the other hand, [Pt(phbpy)(CNR)]A (where A is lipophilic 2,3,4-tris(dodecyloxy)benzene sulfonate) aggregates in non-polar cyclohexane (*λ*_em_ = 673 nm) but exists as a monomer in moderately polar CH_2_Cl_2_ (*λ*_em_ = 530 nm), CHCl_3_ and THF.^16*a*^ A similar behavior was observed for [Pt(N^C^N)(CNR)]Cl with a liphophilic cyclometalated ligand.^20^

Analysis of the solvent-dependent behavior of 1 and 2 reveals that these compounds aggregate following the trend of empirical polarity parameter *E*_T_ (molar transition energy derived from UV-vis CT absorption bands of solvatochromic dyes), which is in the order CH_2_Cl_2_ < acetone < MeCN < MeOH.^46^ This implies that the assembly of sterically unhindered bimetallic cations 1 and 2 can be regulated not only by the concentration and the temperature, but it also distinctly and with unusually high sensitivity responds to the set of subtle intermolecular solvent–solute forces (dipole–dipole, hydrogen bonding, solvophobic interactions, *etc.*).

### Electrochemistry and spectoelectrochemistry (SEC) in solution

Cyclic voltammetry of all four complexes in *n*-Bu_4_NPF_6_/DMF showed two reversible one-electron reduction waves which that we ascribe to ligand-centered processes and one irreversible oxidation wave which we assign to the Pt(ii)/Pt(iii) redox couple (Fig. S19, ESI†) in line with our previous study on [Pt(phbipy)(CN)].^23^ The two reduction waves for complex 1, found at negative potentials (−2.88 and −2.21 V, *vs.* ferrocene/ferrocenium; Table S7, ESI†), are well resolved (*i.e.*, separated by 67 mV), in accordance with the bpy unit being the electron-acceptor.^23,48^ The other three complexes showed less negative potentials with small separations of about 10 mV, and additional irreversible reduction waves at more negative potentials. This reflects that the triazolyl-pyridine (phpytabn), the pyrazolyl-pyridine (phpypz), and the (pyridin-2-yl)pyridin-2-amine (phpyampy) units are superior electron-acceptors compared with bpy but accommodate the second electron in the same unit (presumably triazol, pyrazol, and the peripheral amino-pyridine) compared with bpy that delocalizes the two electrons over the entire bpy unit. This is fully in line with the DFT-calculated contributions to the LUMO and the character of the hole from the natural transition orbital hole-particle calculations (Fig. 5 and S10, ESI†).

The oxidation profile is also very different for the bpy complex 1, if compared with the others. 1 showed a pronounced oxidation wave at around 0 V (*vs.* ferrocene/ferrocenium), the oxidation potentials of the other complexes lie more than 0.9 V higher. This leads to a markedly smaller electrochemical HOMO–LUMO gap for 1 (2.12 eV) compared with 2 to 4 (2.72 to 3.16 eV) (HOMO = highest occupied molecular orbital, LUMO = lowest unoccupied molecular orbital). The same trend was found for the optical HOMO–LUMO gaps (Table S3, ESI†).

SEC was carried out in *n*-Bu_4_NPF_6_/DMF solutions on all four compounds using an optical-transparent-thin-layer-electrochemical cell at 298 K.^49^ The ^1^H NMR spectrum of 1 measured in DMF-*d*_7_ confirms that the diplatinum cations are stable and no dissociation at 298 K is detected.

Upon electrochemical reduction of 1 (*c* = 1 mM), the long-wavelength absorption bands at 570 and 340 nm loose most of their intensity and broad structured bands centering around 900 nm, 550 nm and 360 nm appear (Fig. S20, ESI†). They are typical for the reduced phbpy ligand.^48^ The first electrochemical reduction of 2 produces a similar structured band system peaking around 850 nm, which is replaced by absorption bands with a maximum at 640 nm during a second reduction (Fig. S21, ESI†). These long-wavelength bands likely originate from the reduced phpypz chromophoric group.

Electrochemical reduction of compounds 3 and 4 leads to increased structured absorption bands in the range 400 to 500 nm (Fig. S22 and S23, ESI†). The lack of long-wavelength absorption bands for the reduced species, comparable to those observed for 1 and 2, correlates with the weaker π-acceptor capacity of the cyclometalated ligands in 3 and 4 and higher energies of the π*-levels caused by the smaller contributions of triazine and aminopyridine groups to the phenyl-pyridine chromophore, if compared with pyridine and pyrazine in 1 and 2. This is perfectly in line with the DFT-calculated electronic structures (Fig. S10, ESI,† for 1, see ref. 23), according to which the electronic transitions are mainly localized on the phenyl-pyridine part of the ligands with a marked contribution from the pyridine group in 1 (ref. 23) and pyrazine group in 2 but marginal contributions from triazine and aminopyridine in 3 and 4.

**Fig. 8:**
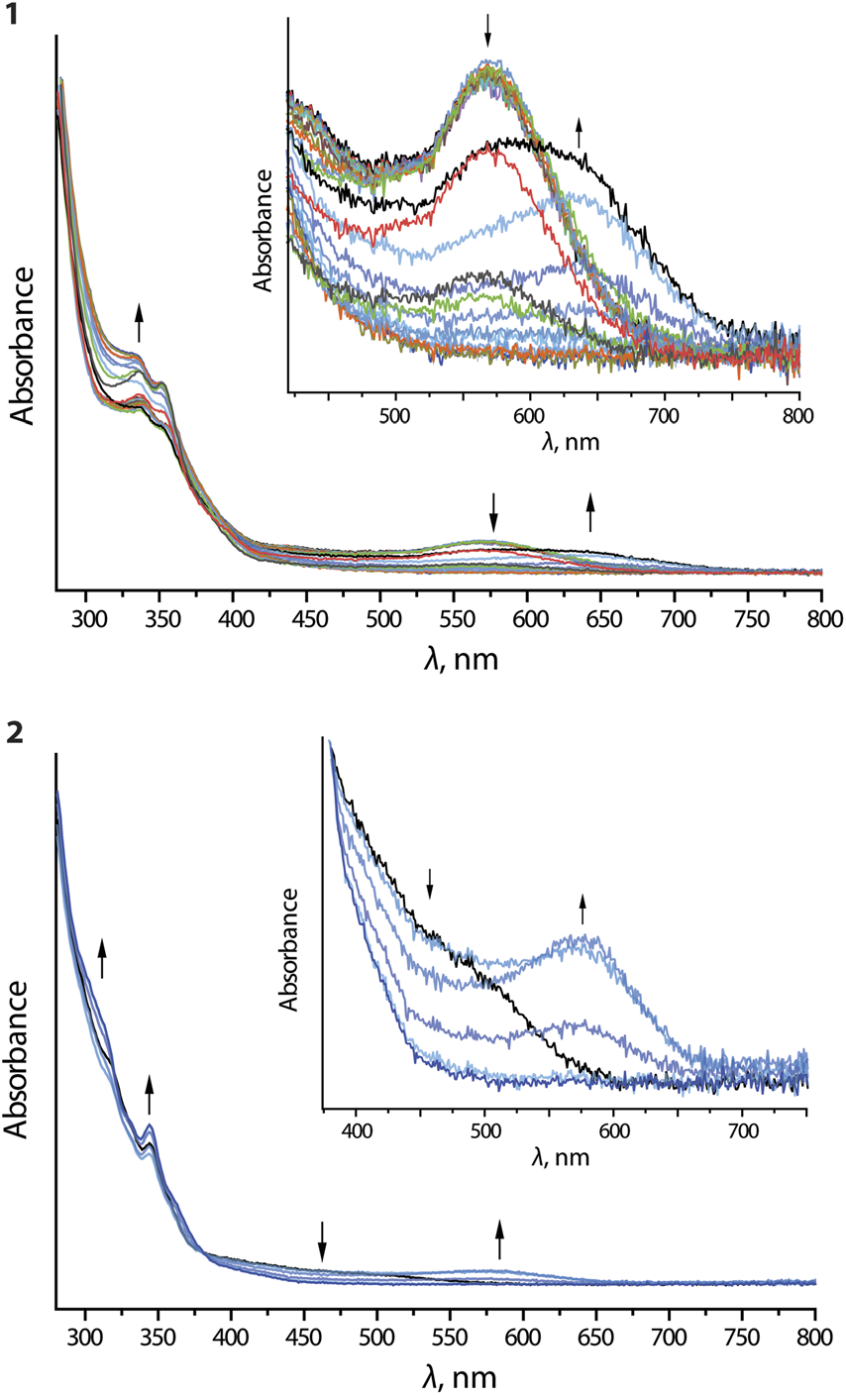
UV-vis absorption spectra recorded during anodic electrolysis (oxidation) of complexes 1 and 2 in *n*Bu_4_NPF_6_/DMF.

Upon oxidation of compounds 1 and 2, the long-wavelength absorption bands at 570 (1) and 500 (2) nm are replaced by broad signals at 640 (1) and 570 (2) nm, respectively (Fig. 8). On prolonged electrolysis, all long-wavelength features are lost and some additional intensity for the bands around 350 nm is generated. The emergence of the new low-energy bands is very probably the result of oxidation of the dimeric assemblies.

The removal of an electron from the antibonding dσ*-orbital stemming from the Pt⋯Pt interaction leads to a contraction of the metal⋯metal distance and thus a decrease in the optical gap. This effect conceptually resembles the MMLCT process, which causes shortening of the intermetallic separations in the excited state. Complexes 3 and 4 do not show significant changes upon oxidation (Fig. S24 and S25, ESI†), presumably because of high oxidation potential and negligible aggregation at 298 K.

## Conclusions

A family of diplatinum complexes [{Pt(C^N^^/^*N)}_2_(CN)][BAr_4_^F^] bearing tridentate cyclometalated ligands were prepared by utilizing a cyanido unit as the bridging ligand. These compounds are highly phosphorescent under ambient conditions reaching quantum yields up to 0.73 in solution (3, HC^N^N = (benzyltriazolyl)-phenylpyridine) and 0.62 in the solid state (3). Complexes 1 to 3 in their discrete molecular form show visibly higher efficiencies than their mononuclear analogues [Pt(C^N^^/^*N)(CN)]. In the absence of steric hindrance, *i.e.* in the case of HC^N^N = phenyl-2,2′-bipyridine (1) and pyrazolyl-phenylpyridine (2) ligands, the cationic complexes in 1 and 2 reveal a pronounced tendency towards aggregation in solution, which distinctly depends not only on the concentration and temperature, but also on solvent polarity (CH_2_Cl_2_ < acetone < MeCN < MeOH). The formation of supramolecular structures was evaluated for 1 by diffusion NMR spectroscopy, which indicated that the assembly of tetrameric species [{Pt(phbpy)}_2_(CN)]_4_^4+^ can be reached in acetone. In line with the NMR studies, the concentration and solvent-regulated aggregation of 1 and 2 is manifested by the changes in optical properties. In particular, the increase of solvent polarity from CH_2_Cl_2_ to MeOH causes gradual bathochromic shift of the emission from green to the NIR region of the spectrum assigned to the transition from the monomer to the trimer or larger aggregates. Despite the bulky counterion ^−^BAr_4_^F^, complexes 1 and 2 undergo extensive association in the solid state *via* Pt⋯Pt and π–π stacking interactions resulting in low-energy emission with the maximum up to *λ*_em_ = 912 nm (1).

The presented study shows that the rigid bimetallic architecture composed of conventional Pt(ii) luminescent units can improve the photophysical performance in terms of quantum efficiency. Importantly, the dramatic enhancement of the aggregation ability of diplatinum cations induces unusual sensitivity to dipole–dipole, hydrogen bonding, and solvophobic interactions, *i.e.* the properties of the surrounding medium. This substantially expands the borders of optical dynamic behavior of responsive molecular Pt(ii) complexes.

## Data availability

Experimental procedures, spectroscopic and crystallographic data, optimized structures of the aggregates, are available in the ESI† of this article.

## Author contributions

A. K., C. S. and I. O. K. directed the project. V. K. synthesized the compounds and carried out crystallographic and NMR characterization together with N. K. and I. O. K., S. B. performed photophysical studies. T. E. and P. H. were responsible for computational analysis. J. F., L. K. and A. K. carried out electrochemical and spectroelectrochemical experiments. All authors participated in the interpretation of the results and writing the manuscript.

## Conflicts of interest

There are no conflicts to declare.

## Supplementary Material

SC-015-D3SC06941A-s001

SC-015-D3SC06941A-s002

SC-015-D3SC06941A-s003
