# Supplementary material for: Cyanido-bridged diplatinum(ii) complexes: ligand and solvent effect on aggregation and luminescence
Source: Chem Sci. 2024 Feb 12;15(11):4005–18. doi: 10.1039/d3sc06941a (PMC10935663; doi:10.1039/d3sc06941a)
Supplement: SC-015-D3SC06941A-s001 [file SC-015-D3SC06941A-s001.pdf]

Electronic Supplementary Material (ESI) for Chemical Science.
This journal is © The Royal Society of Chemistry 2024

Electronic Supporting Information (ESI)

Cyanido-bridged diplatinum(II) complexes: ligand and solvent effect on aggregation and luminescence

Viktoria V. Khistiaeva,[a] Stefan Buss,[b] Toni Eskelinen,[a] Pipsa Hirva,[a] Niko Kinnunen,[a] Joshua Friedel,[c] Lukas Kletsch,[d] Axel Klein,[c] Cristian A. Strassert,*[b] Igor O. Koshevoy*[a]*

[a] *Department of Chemistry, University of Eastern Finland, P.O. Box 111, FI-80100 Joensuu, Finland.*

[b] *Institut für Anorganische und Analytische Chemie, Universität Münster, CiMIC, CeNTech, Heisenbergstraße 11, 48149 Münster, Germany*

[c] *University of Cologne, Faculty of Mathematics and Natural Sciences, Department of Chemistry, Institute for Inorganic Chemistry, Greinstrasse 6, D-50939 Cologne, Germany.*

E-mail: axel.klein@uni-koeln.de, cstra_01@uni-muenster.de, igor.koshevoy@uef.fi

Contents

Experimental Section S3

Supporting Tables and Figures S7

Table S1. Crystal data and structure refinement for **1**, **2**, **3**·CH_2Cl_2 and **4**. S8

Table S2. Selected bond lengths (Å) and angles (°) for complexes **1**, **3**·CH_2Cl_2 and **4**. S10

Fig. S1. Molecular view and fragment of crystal packing of complex **2**. S11

Fig. S2. Fragments of crystal packing for complexes **1**, **3**·CH_2Cl_2, and **4.** S12

Fig. S3. Powder XRD patterns for **1** to **4**. S13

Fig. S4. Photoluminescence spectra of compounds **1** to **4** in the solid state at 77 K. S14

Fig. S5. Photoluminescence spectra of complex **3** in the solid state at 298 K. S14

Fig. S6. ESI-MS of compounds **1** to **4**. S15

Fig. S7, ^{1}H NMR spectra of compounds **1** and **2** at 293 K. S16

Fig. S8. ^{1}H NMR spectra of compounds **3** and **4** at 293 K. S17

Fig. S9. UV-vis absorption spectra of compounds **1** to **4**. S18

Table S3. UV-vis absorption maxima of compounds **1** to **4**. S18

Table S4. TD-DFT-calculated lowest-lying singlet-singlet vertical excitations and lowest singlet- S19

triplet vertical excitations for diplatinum cations of **1** to **4** in CH_2Cl_2.

Fig. S10. Natural transition orbital hole-particle pair for the vertical $S_0 \rightarrow S_1$ transitions for the diplatinum cations of **2** to **4** in CH_2Cl_2. S20

Table S5. Photophysical data of mononuclear congeners [Pt(C^N^/*N)(CN)] (**1_mono** to **4_mono**) in CH_2Cl_2 solution. S20

Fig. S11. Excitation and emission spectra of complexes [Pt(C^N^/*N)(CN)] (**1_mono** to **4_mono**) in solution. S21

Fig. S12. Natural transition orbital hole-particle pair for the vertical $T_1 \rightarrow S_0$ transitions for the diplatinum cations of **2** to **4**. S22

Table S6. DFT-predicted vibrationally resolved phosphorescence for complexes **1** to **4** and their aggregates. S22

Fig. S13. Room temperature emission spectra of **1** in acetone. S23

Fig. S14. DFT-optimized ground state geometries of the dimers $[\mathbf{1}]_2^{2+}$ and the trimer $[\mathbf{1}]_3^{3+}$, natural transition orbital hole-particle pairs for the $T_1 \rightarrow S_0$ transitions. S24

Figure S15. Time-resolved photoluminescence decay profiles for **2** in CH_2Cl_2, acetone, MeCN, MeOH (c = 1 mM, room temperature). S25

Fig. S16. DFT-optimized ground state geometries of the dimers $[\mathbf{2}]_2^{2+}$ and the trimer $[\mathbf{2}]_3^{3+}$, natural transition orbital hole-particle pairs for the $T_1 \rightarrow S_0$ transitions. S26

Fig. S17. Emission spectra of optically dilute **1** to **4** in frozen (77 K) CH_2Cl_2:MeOH 1:1 v/v mixture. S27

Fig. S18. DTF-optimized ground state geometry of the dimer $[\mathbf{3}]_2^{2+}$, natural transition orbital hole-particle pairs for the $T_1 \rightarrow S_0$ transition. S27

Fig. S19. Cyclic voltammograms of **1** to **4** in n-Bu_4NPF_6/DMF at a scan rate of 100 mV/s at 298 K. S28

Table S8. Redox potentials of the Pt(II) complexes **1** to **4** in n-Bu_4NPF_6/DMF. S28

Figs. S20–S23. UV-vis absorption spectra recorded during cathodic electrolysis (reduction) of compounds **1** to **4** in n-Bu_4NPF_6/DMF. S29, S30

Figs. S24, **S25.** UV-vis absorption spectra recorded during anodic electrolysis (oxidation) of compounds **3** and **4** in n-Bu_4NPF_6/DMF. S31

References S32

Experimental Section

Materials. [Pt(phbpy)Cl] (Hphbpy = 6-phenyl-2,2'-bipyridine),[1] [Pt(phpytabn)Cl] (Hphpytabn = 2-(1-benzyl-1H-1,2,3-triazol-4-yl)-6-phenylpyridine),[2] [Pt(phpypz)Cl] (Hphpypz = 2-phenyl-6-(1H-pyrazol-1-yl)pyridine),[3] [Pt(phpy5apy)Cl] (Hphpyampy = *N*-pentyl-6-phenyl-*N*-(pyridin-2-yl)pyridin-2-amine)[4] were prepared according to published procedures. Other reagents and solvents were used as received.

Syntheses

General procedure for the preparation of the compounds [{Pt(C^N^/*N)}$_2$(CN)][BAr$^F{}_4$]. [Pt(C^N^/*N)Cl] (0.22 mmol) was dissolved in DMSO (15 mL) and Na[BAr$^F{}_4$] (197 mg, 0.22 mmol) was added. The mixture was stirred under nitrogen for one hour, then it was diluted with CH_2Cl_2 (10 mL) and water (5 mL), the stirring was continued overnight in inert atmosphere. The intermediate complex [Pt(C^N^/*N)(DMSO)][BAr$^F{}_4$] was extracted with CH_2Cl_2 (3×10 mL), the organic phase was washed with water (4×10 mL), dried over anhydrous Na_2SO_4, filtered and evaporated. The resulting orange oil was dissolved in MeCN (10 mL), degassed and a solution of NaCN (5.2 mg, 0.11 mmol) in MeOH (2 mL) was added dropwise under a nitrogen atmosphere. The reaction mixture was stirred under nitrogen overnight. The solvents were removed, the crude product was purified by crystallization.

[{Pt(phbpy)}$_2$(CN)][BAr$^F{}_4$] (1). Prepared from [Pt(phbpy)]Cl. Recrystallization by slow evaporation of a MeOH-toluene solution of **1** at room temperature gave dark violet microcrystalline material (91%). Single crystals suitable for XRD analysis were obtained by slow evaporation of a CH_2Cl_2 solution of **1**. ESI-MS (*m/z*): $[M]^+$ 878.1171 (calcd. 878.1171). ^{1}H NMR (500 MHz, CD_2Cl_2, 0.8 mM, 298 K) δ (ppm): 8.96 (dm br, J_{HH} 5.6 Hz, py, 1H), 8.89 (dm br, J_{HH} 5.6 Hz, py, 1H), 8.20 (td, J_{HH} 7.9 and 1.5 Hz, py, 1H), 8.05 (dd, J_{HH} 7.9 and 4.0 Hz, py, 2H), 8.02 (t, J_{HH} 8.1 Hz, py, 1H), 7.98 (t, J_{HH} 8.1 Hz, py, 1H), 7.74–7.62 (m, BAr$^F{}_4$ + Ph/py, 15H), 7.58 (dd, J_{HH} 7.5 and 1.3 Hz, py, 1H), 7.55 (s br, BAr$^F{}_4$, 4H), 7.50–7.45 (m, 2H), 7.24 (td, J_{HH} 7.4 and 1.5 Hz, py, 1H), 7.20–7.15 (m, 3H). ^{13}C{^{1}H} NMR (100.6 MHz, acetone-d_6, 300 K, c = 10 mM) δ (ppm): 166.1, 164.8, 163.5, 163.0, 162.5, 162.0, 156.2, 155.6, 155.5, 155.3, 152.3, 149.4, 146.3, 146.1, 143.2, 142.0, 141.7, 141.1, 140.8, 137.6, 135.7 (m, $^-$BAr$^F{}_4$), 134.2, 133.4, 132.1, 131.6, 130.2 (qm, J_{CF} 31.6 Hz, $^-$BAr$^F{}_4$), 129.6, 128.4, 128.1, 126.9, 126.5, 126.1, 125.0, 124.9, 124.7, 124.4, 124.2, 121.5, 120.1, 119.9, 119.7, 119.6, 118.6 (m, $^-$BAr$^F{}_4$),. ^{195}Pt{^{1}H} NMR (86 MHz, acetone-d_6, 300 K, c = 10 mM) δ (ppm): -3902 and -3534. Anal. Calcd for $C_{65}H_{34}BF_{24}N_5Pt_2$: C, 44.82; H, 1.97; N 4.02. Found: C, 44.80; H, 2.04; N 4.02.

[{Pt(phpypz)}$_2$(CN)][BAr$^F{}_4$] (2). Prepared from [Pt(phpypz)]Cl. Recrystallization by slow evaporation of a CH_2Cl_2/EtOH (1:1 v/v mixture) solution of **2** at room temperature gave a bright red

microcrystalline material (78%). ESI-MS (*m/z*): $[M]^+$ 856.1051 (calcd. 856.1076). ^1H NMR (500 MHz, CD_2Cl_2, 0.7 mM, 298 K) δ (ppm): 8.28 (t, J_{HH} 2.6 Hz, pz, 2H), 8.15–8.07 (m, py, 4H), 7.75 (m, 1H), 7.72 (m, BAr^F_4, 8H), 7.69 (d, J_{HH} 8.0 Hz, 1H), 7.62 (d, J_{HH} 8.0 Hz, 1H), 7.61–7.58 (m, 2H), 7.55 (m br, BAr^F_4+ph, 5H), 7.41 (d, J_{HH} 8.3 Hz, py, 1H), 7.35 (d, J_{HH} 8.3 Hz, py, 1H), 7.29 (m, 1H), 7.26–7.23 (m, 3H), 6.94 (dd, J_{HH} 3.0 and 2.2 Hz, pz, 1H), 6.89 (dd, J_{HH} 2.9 and 2.2 Hz, pz, 1H). $^{13}C\{^1H\}$ NMR (125.7 MHz, acetone-d_6, 300 K, c = 9 mM) δ (ppm): 164.9, 163.6, 163.3, 162.9, 162.5, 162.1, 149.1, 148.4, 146.0, 145.6, 145.4, 144.5, 143.8, 142.7, 140.7, 140.8, 138.1, 135.6 (m, $^-BAr^F_4$), 134.4, 132.6, 132.4, 132.0, 131.4, 130.1 (qm, J_{CF} 31.6 Hz, $^-BAr^F_4$), 128.7, 126.7, 126.6, 126.3, 124.9, 124.8, 124.4, 122.2, 118.6 (m, $^-BAr^F_4$), 117.0, 116.7, 112.0, 111.7, 108.3, 108.0. $^{195}Pt\{^1H\}$ NMR (86 MHz, acetone-d_6, 300 K, c = 6 mM) δ (ppm): -3991 and -3579. Anal. Calcd for $C_{61}H_{32}BF_{24}N_7Pt_2$: C, 42.60; H, 1.88; N 5.70. Found: C, 42.04; H, 1.84; N 5.72.

[{Pt(phpytabn)}$_2$(CN)][BAr$^F_4$] (3). Prepared from [Pt(phpytabn)]Cl. Recrystallization by gas-phase diffusion of pentane into a CH_2Cl_2/MeOH (10:1 v/v mixture) solution of **3** gave yellow crystals (87%). ESI-MS (*m/z*): $[M]^+$ 1038.1872 (calcd. 1038.1920). ^1H NMR (500 MHz, acetone-d_6, 298 K) δ (ppm): 8.73 (s, triazole, 1H), 8.72 (s, triazole, 1H), 7.99 (dd, J_{HH} 7.6 and 1.0 Hz, py, 1H), 7.98 (t, J_{HH} 7.9 Hz, py, 1H), 7.94 (t, J_{HH} 8.0 Hz, py, 1H), 7.84 (dd, J_{HH} 7.6 and 1.0 Hz, py, 1H), 7.79 (m, BAr^F_4, 8H), 7.67 (s, BAr^F_4, 4H), 7.65 (d, J_{HH} 6.2 Hz, Ph, 1H), 7.62 (d, J_{HH} 8.8 Hz, Ph, 1H), 7.60–7.56 (m, 6H), 7.50–7.42 (m, 6H), 7.40 (dd, J_{HH} 7.7 and 1.2 Hz, Ph, 1H), 7.38 (dd, J_{HH} 7.7 and 1.1 Hz, Ph, 1H), 7.25 (td, J_{HH} 7.4 and 1.4 Hz, Ph, 1H), 7.20 (td, J_{HH} 7.4 and 1.4 Hz, Ph, 1H), 7.07 (td, J_{HH} 7.5 and 1.2 Hz, Ph, 1H), 7.06 (td, J_{HH} 7.5 and 1.2 Hz, Ph, 1H), 5.83 (s, 2H, CH_2), 5.82 (s, 2H, CH_2). $^{13}C\{^1H\}$ NMR (125.7 MHz, acetone-d_6, 298 K) δ (ppm): 166.9, 165.7, 163.3, 162.9, 162.5, 162.1, 151.8, 150.5, 149.7, 149.1, 147.0, 146.6, 142.9, 141.9, 141.0, 140.4, 139.8, 136.0, 135.6 (m, $^-BAr^F_4$), 134.8, 134.7, 132.2, 131.6, 130.2, 130.1 (qm, J_{CF} 31.6 Hz, $^-BAr^F_4$), 129.9, 129.8, 128.7, 126.5, 126.4, 126.3, 125.8, 125.4, 125.3, 125.2, 124.4, 122.2, 118.8, 118.6 (m, $^-BAr^F_4$), 118.5, 118.3, 56.7. $^{195}Pt\{^1H\}$ NMR (86 MHz, acetone-d_6, 300 K) δ (ppm): -4019 and -3614. Anal. Calcd. for $C_{73}H_{42}BF_{24}N_9Pt_2$: C, 46.10; H, 2.23; N 6.63. Found: C, 46.05; H, 2.16; N 6.65.

[{Pt(phpy5apy)}$_2$(CN)][BAr$^F_4$] (4). Prepared from [Pt(phpy5apy)Cl]. The crude product was extracted with toluene (5 × 5 mL) and the solvent was removed. Recrystallization by slow evaporation of an EtOH/water (3:1 v/v mixture) solution of **4** at room temperature gave bright yellow-green crystals (74%). ESI-MS (*m/z*): $[M]^+$ 1048.3006 (calcd. 1048.2954). ^1H NMR (500 MHz, acetone-d_6, 298 K) δ (ppm): 9.27 (ddd, J_{HH} 6.0, 1.8 and 0.4 Hz, py, 1H), 9.22 (ddd, J_{HH} 5.9, 1.8 and 0.4 Hz, py, 1H), 8.30 (td, J_{HH} 7.5 and 1.8 Hz, Ph, 1H), 8.29 (td, J_{HH} 7.6 and 1.9 Hz, Ph, 1H), 8.25 (dd, J_{HH} 8.0 and 7.6 Hz, Ph, 1H), 8.20 (dd, J_{HH} 8.0 and 7.6 Hz, Ph, 1H), 7.97 (d, J_{HH} 8.2 Hz, Ph, 1H), 7.93–7.90 (m, 3H), 7.85–7.81 (m, 3H), 7.80–7.76 (m, BAr^F_4 + Ph, 9H), 7.69–7.5 (m, BAr^F_4 + Ph, 5H), 7.56 (dd,

J_{HH} 8.4 and 0.9 Hz, Ph, 1H), 7.42 (ddd, J_{HH} 7.1, 5.9 and 1.2 Hz, py, 1H), 7.37 (ddd, J_{HH} 7.2, 5.9 and 1.2 Hz, py, 1H), 7.22–7.17 (m, 2H), 7.15 (td, J_{HH} 7.4 and 1.5 Hz, py, 1H), 7.11 (td, J_{HH} 7.4 and 1.4 Hz, py, 1H), 4.41 (m, CH_2, 4H), 1.70 (m, CH_2, 4H), 1.38 (m, CH_2, 4H), 1.24 (m, CH_2, 4H), 0.81 (m, CH_3, 6H). $^{13}C\{^1H\}$ NMR (125.7 MHz, acetone-d_6, 298 K) δ (ppm): 167.2, 165.7, 163.3, 162.9, 162.5, 162.1, 154.6, 154.2, 153.9, 153.4, 153.3, 149.4, 146.2, 142.8, 141.9, 141.8, 141.6, 141.2, 139.9, 135.6 (m, $^{-}BAr^F_4$), 135.2, 131.2, 130.5, 130.1 (qm, J_{CF} 31.6 Hz, $^{-}BAr^F_4$), 128.7, 126.6, 126.0, 125.8, 125.5, 125.0, 124.4, 122.3, 121.8, 120.9, 120.3, 118.6 (m, $^{-}BAr^F_4$), 116.6, 116.5, 114.9, 114.8, 55.0, 29.3, 28.5, 22.8, 14.4. $^{195}Pt\{^1H\}$ NMR (86 MHz, acetone-d_6, 300 K) δ (ppm): –3852 and –3457. Anal. Calcd. for $C_{75}H_{56}BF_{24}N_7Pt_2$: C, 47.11; H, 2.95; N 5.13. Found: C, 46.29; H, 2.70; N 4.97.

Instrumentation

The solution ^1H, $^{13}C\{^1H\}$, $^{195}Pt\{^1H\}$ NMR spectra were recorded on a Bruker Avance III 400 and a JEOL-500 MHz spectrometers. CHN Microanalyses were carried out on an Elementar Vario Micro Cube instrument at the analytical laboratory of the University of Eastern Finland.

Electrochemistry. Cyclic voltammetry measurements were carried out in 0.1 M nBu$_4$NPF$_6$/DMF solution at 298 K and at 100 mV/s scan rate, using a three-electrode configuration (glassy carbon working electrode, Pt counter electrode, Ag/AgCl pseudo-reference electrode) and a Metrohm µStat400 potentiostat. The potentials were referenced against the ferrocene/ferrocenium redox couple as internal standard. UV-vis-spectroelectrochemical measurements (in 0.1 M nBu$_4$NPF$_6$/DMF solution, at 298 K) were performed using an optically transparent thin-layer electrode (OTTLE) cell,[5] a Cary 50 scan spectrophotometer, and a Metrohm Autolab PGSTAT30 potentiostat and function generator.

X-ray diffractometric structural analysis. The crystals of **1**, **2**, **3**·CH_2Cl_2 and **4**, were collected from CH_2Cl_2, pentane/CH_2Cl_2/MeOH, and EtOH/water solutions, respectively. They were immersed in cryo-oil, mounted in a Nylon loop, and measured at T = 150 K. The X-ray diffraction data were collected with a Bruker Kappa Apex II diffractometer using Mo Kα (λ = 0.71073 Å) radiation. The APEX[6] program package was used for cell refinements and data reductions. A numerical or semiempirical absorption correction (SADABS)[7] was applied to all data. The structures were solved by direct methods using the SHELXS-2018[8] program with the WinGX[9] graphical user interface. Structural refinements were carried out using SHELXL-2018.[8]

The diplatinum cations of **1** and **2** were disordered between two close positions and modeled with occupancies of 0.49/0.51 and 0.44/0.56. The crystallographic disorder is likely accompanied by

chemical disorder (i.e. two possible orientations of cyclometalated C^N^N and cyanide ligands). Due to these features, a series of geometry and displacement constraints and restraints were applied to the ligands. In addition, several CF_3 groups of the borate anion were disordered too.

In **3**·CH_2Cl_2 and **4**, the carbon and nitrogen atoms of the cyanide group were refined with occupancies of 0.5 being placed in the same position.

All non-H atoms were anisotropically refined, and all hydrogen atoms were positioned geometrically and constrained to ride on their respective parent atoms with C−H = 0.95−0.99 A and *U*iso = 1.2−1.5*U*equiv (parent atom). The crystallographic details are summarized in Table S1.

Photophysical studies. Absorption spectra were measured with a Shimadzu UV-3600 I plus UV-vis-NIR spectrophotometer. For all absorption measurements, matched quartz (Hellma®) cuvettes were used.

Steady-state excitation and emission spectra were recorded on a FluoTime 300 spectrometer from PicoQuant equipped with a 300 W ozone-free Xe lamp (250–900 nm), a 10 W Xe flash-lamp (250–900 nm, pulse width *ca.* 1 μs) with repetition rates of 0.1 to 300 Hz, a double-grating excitation monochromator (Czerny-Turner type, grating with 1200 lines/mm, blaze wavelength: 300 nm), diode lasers (pulse width < 80 ps) operated by a computer-controlled laser driver PDL-828 "Sepia II" (repetition rate up to 80 MHz, burst mode for slow and weak decays), two double-grating emission monochromators (Czerny-Turner, selectable gratings blazed at 500 nm with 2.7 nm/mm dispersion and 1200 lines/mm, or blazed at 1200 nm with 5.4 nm/mm dispersion and 600 lines/mm) with adjustable slit width between 25 μm and 7 mm, Glan-Thompson polarizers for excitation (after the Xe-lamps) and emission (after the sample). Different sample holders (Peltier-cooled mounting unit ranging from –15 to 110 °C or an adjustable front-face sample holder), along with two detectors (namely a PMA Hybrid-07 from PicoQuant with transit time spread FWHM < 50 ps, 200–850 nm, or a H10330C-45-C3 NIR detector with transit time spread FWHM 0.4 ns, 950-1700 nm from Hamamatsu) were used. Steady-state spectra and photoluminescence lifetimes were recorded in the TCSPC mode by a PicoHarp 300 (minimum base resolution 4 ps) or in the MCS mode by a TimeHarp 260 (where up to several ms can be traced). Emission and excitation spectra were corrected for source intensity (lamp and grating) by standard correction curves. For samples with lifetimes in the ns order, an instrument response function calibration (IRF) was performed using a diluted Ludox® dispersion. Lifetime analysis was performed using the commercial EasyTau 2 software (PicoQuant). The quality of the fit was assessed by minimizing the reduced chi squared function (χ^2) and visual inspection of the weighted residuals and their autocorrelation. All solvents used were of spectrometric grade (Uvasol®, Merck).

Photoluminescence (PL) quantum yields were measured with a Hamamatsu Photonics absolute PL quantum yield measurement system (C9920-02) equipped with a L9799-01 CW Xe light source (150 W), a monochromator, a C7473 photonic multi-channel analyzer, an integrating sphere and employing U6039-05 software (Hamamatsu Photonics, Ltd., Shizuoka, Japan).

Uncertainties of k_r and k_{nr} were calculated as

$$\Delta k_R = \left(\frac{\Delta \Phi_{L(avgd)}}{\tau_{amp(avgd)}}\right) + \left(\frac{\Delta \tau_{amp(avgd)}}{\tau_{amp(avgd)}^{2}} \Phi_{L(avgd)}\right)$$

$$\Delta k_{NR} = \left(\frac{\Delta \tau_{amp(avgd)}}{\tau_{amp(avgd)}^{2}}\right) + \left(\frac{\Delta \Phi_{L(avgd)}}{\tau_{amp(avgd)}}\right) + \left(\frac{\Delta \tau_{amp(avgd)}}{\tau_{amp(avgd)}^{2}} \Phi_{L(avgd)}\right).$$

For measurements of fluid solutions (diluted optically to A < 0.1) and frozen glassy matrices, round cuvettes made from quartz were used. The solids were measured using quartz NMR tubes.

Computational details. Single molecule models for cations **1–4** were optimized in the ground state and first excited triplet state at the (TD-)DFT level of theory. Optimizations for aggregated models were carried out using coordinates from crystallographically determined structures with a QM/MM approach.[10] In the QM/MM optimizations, the crystal structures were expanded in three dimensions and one dimer from the middle of the supercell was assigned as the QM part and treated with DFT, while the surroundings were treated with a simple force field (UFF)[11] and kept frozen during the optimization. In addition, free optimization of dimeric and trimeric aggregates of **1** and **2** starting from head-to-tail orientation of cyclometalated fragments was performed. Triplet state structures of aggregated models were optimized with unrestricted DFT formalism. Single-point TD-DFT calculations were performed for each of the optimized structures to study the excitation/emission behavior. All calculations were done with the Gaussian 16 program package.[12] The meta-hybrid MN15[13] functional was used for all calculations, combined with the def2-TZVPP basis set, and the corresponding ECP (effective core potential) for platinum atoms, whereas lighter atoms were treated with the def2-SVP basis set.[14] The conductor-like polarizable continuum model (C-PCM)[15] with CH_2Cl_2 solvation was used to describe the solvent effects. Vibrationally resolved phosphorescence spectra were also calculated for the monomeric models at the Franck-Condon limit with exact Hessians calculated for both the ground and excited state (AdiabaticHessian mode). Time-Dependent formalism was used to calculate the band shape with a temperature of 298 K.

Supporting Tables and Figures

Table S1. Crystal data and structure refinement for **1**, **2**, **3**$\cdot CH_2Cl_2$ and **4**.

	**1**	**2**	**3**$\cdot CH_2Cl_2$	**4**
CCDC	2285471	2285472	2285473	2285474
Empirical formula	$C_{130}H_{68}B_2F_{48}N_{10}Pt_4$	$C_{61}H_{32}BF_{24}N_7Pt_2$	$C_{74}H_{44}BCl_2F_{24}N_9Pt_2$	$C_{150}H_{112}B_2F_{48}N_{14}Pt_4$
Formula weight	3483.92	1719.92	1987.07	3824.51
Temperature (K)	150(2)			
Wavelength (Å)	0.71073			
Crystal system	monoclinic	monoclinic	triclinic	triclinic
Space group	*C*2/*c*	*C*2/*c*	*P* 1	*P* 1
Unit cell dimensions				
a (Å)	37.877(4)	37.583(4)	10.8326(5)	19.0654(6)
b (Å)	19.7175(19)	19.550(2)	18.8796(9)	21.3165(7)
c (Å)	16.623(2)	16.6042(19)	20.8423(11)	22.1528(8)
a (°)	90	90	63.426(2)	61.9080(10)
β (°)	102.157(11)	103.385(2)	78.863(2)	86.4950(10)
γ (°)	90	90	73.683(2)	65.2190(10)
Volume ($Å^3$)	12136(2)	11869(2)	3647.4(3)	7106.0(4)
Z	4	8	2	2
ρ_{calc} (Mg/m^3)	1.907	1.925	1.809	1.787
μ (mm^{-1})	4.727	4.833	4.017	4.046
F(000)	6688	6592	1924	3728
Crystal size (mm^3)	0.310 × 0.252 × 0.136	0.367 × 0.292 × 0.162	0.703 × 0.263 × 0.106	0.411 × 0.349 × 0.244
θ range for data collection (°)	2.119 to 28.929	2.119 to 25.999	1.965 to 35.00	2.146 to 28.000
Index ranges	-50<=h<=47, -26<=k<=25, -21<=l<=21	-46<=h<=46, -24<=k<=24, -20<=l<=20	-12<=h<=17, -30<=k<=28, -33<=l<=30	-25<=h<=25, -27<=k<=28, -29<=l<=29
Reflections collected	83879	39691	110532	128978
Independent reflections	15218	11622	32025	34259
R_{int}	0.0311	0.0301	0.0238	0.0232
Completeness to θ = 25.24°	99.9%	99.7%	99.9%	99.8%
Absorption correction	Numerical			
Max. and min. transmission	0.566 and 0.322	0.508 and 0.270	0.675 and 0.165	0.438 and 0.287

Data/ restraints/ parameters	15218 / 213 / 974	11622 / 348 / 903	32025 / 60 / 1065	34259 / 192 / 2041
GOOF on F^2	1.284	1.045	1.007	1.027
Final R indices [I>2σ(I)][a]	R1 = 0.0719, wR2 = 0.1455	R1 = 0.0817, wR2 = 0.1870	R1 = 0.0260, wR2 = 0.0605	R1 = 0.0290, wR2 = 0.0638
R indices (all data)	R1 = 0.0897, wR2 = 0.1514	R1 = 0.1150, wR2 = 0.2085	R1 = 0.0393, wR2 = 0.0662	R1 = 0.0429, wR2 = 0.0713
Largest diff. peak and hole (e.Å^{-3})	2.322 and -2.925	4.563 and -2.840	1.756 and -1.839	2.146 and -1.780
[a] $R_1 = \Sigma||F_o| - |F_c||/\Sigma|F_o|$; $wR2 = [\Sigma [w(F_o^2 - Fc^2)^2]/ \Sigma[w(F_o^2)^2]]^{1/2}$				

Table S2. Selected bond lengths (Å) and angles (°) for complexes **1**, **3**$\cdot CH_2Cl_2$ and **4**.

	**1**	**3**$\cdot CH_2Cl_2$	**4**
**Bond lengths, Å**			
Pt–C_{CN}(1)	2.05(5)<br>1.97(7)	1.9440(19)<br>1.9452(17)	1.953(3)<br>1.955(3)<br>1.949(3)<br>1.961(3)
Pt–N_{CN}(1)	1.99(6)<br>1.95(3)	1.9440(19)<br>1.9452(17)	1.953(5)<br>1.955(4)<br>1.949(3)<br>1.961(3)
C(1)–N(1)	1.07(4)<br>1.17(6)	1.152(4)<br>1.150(3)	1.156(7)<br>1.155(6)<br>1.160(5)
Pt(1)–C(2)	2.022(8)<br>2.01(3)<br>2.052(10)<br>2.052(8)	1.986(2)<br>1.989(2)	1.993(4)<br>1.980(4)<br>1.990(4)<br>1.983(4)
Pt(1)–N(2)	1.908(7)<br>1.94(2)<br>1.923(8)<br>1.944(7)	1.9766(17)<br>1.9738(15)	2.009(3)<br>2.007(3)<br>1.997(3)<br>2.010(3)
Pt(1)–N(3)	2.110(8)<br>2.102(14)<br>2.083(10)<br>2.085(8)	2.1034(17)<br>2.0892(17)	2.093(3)<br>2.105(3)<br>2.102(3)<br>2.097(3)
Pt–Pt	3.1925(18)<br>3.2575(30)<br>3.4168(26)<br>3.7064(27)	3.2944(4)	3.6753(6)<br>3.8157(6)
	**Bond angles, °**		
N(1)–C(1)–Pt(1)<br>C(1)–N(1)–Pt(2)	169(3)<br>174(5)<br>168(4)<br>178(5)	177.8(3)<br>178.8(2)	173.1(5)<br>175.1(4)<br>178.1(3)<br>170.9(3)
C(1)–Pt(1)–N(2)<br>N(1)–Pt(2)–N(4)	174.9(11)<br>177(2)<br>175.7(12)<br>175.9(13)	177.36(8)<br>179.15(7)	170.56(14)<br>172.41(13)<br>175.43(12)<br>170.36(13)
C(2)–Pt(1)–N(3)	159.8(4)<br>160.7(8)<br>159.5(6)<br>159.2(4)	161.04(8)<br>160.88(7)	167.54(15)<br>168.59(13)<br>168.72(14)<br>166.65(13)

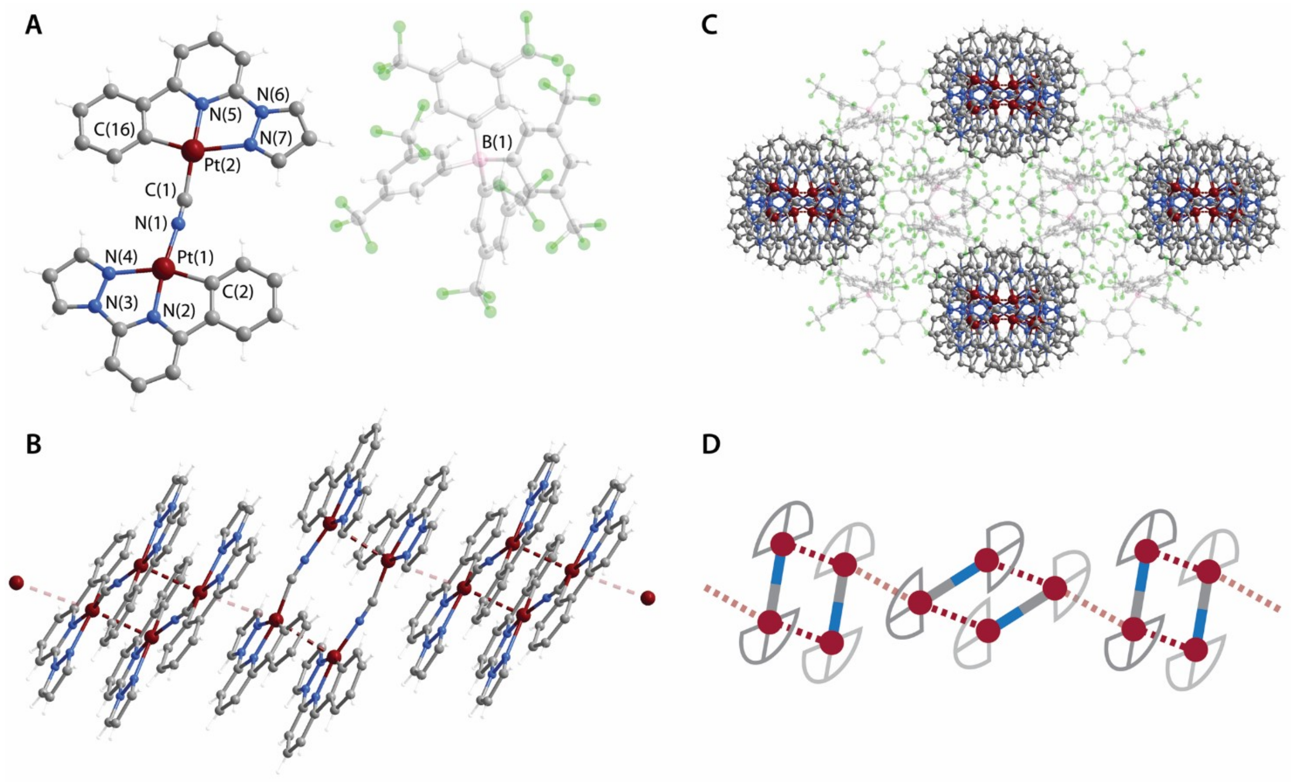

Fig. S1. (A) Molecular view of complex **2** (ball and stick mode, one of the disordered components); (B, D) depiction of the columnar stacks formed by the cations; (C) fragment of crystal packing of **2**, the view is along the columnar stacks of the cations (*c* direction).

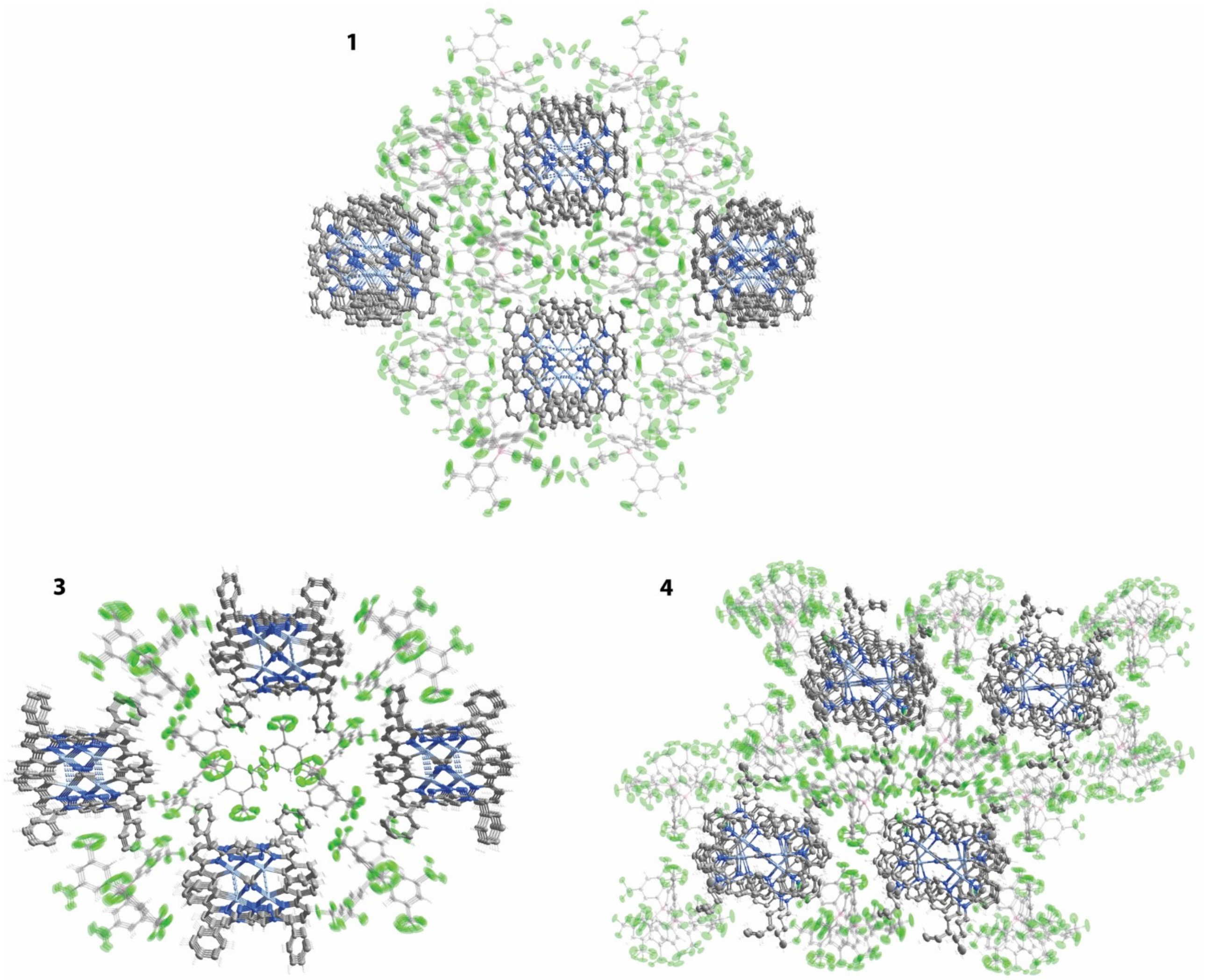

Fig. S2. Fragments of crystal packing for compounds **1**, **3**$\cdot CH_2Cl_2$, and **4**, the views are along the columnar stacks of the cations (*c* direction for **1**, *a* direction for **3**$\cdot CH_2Cl_2$, and **4**).

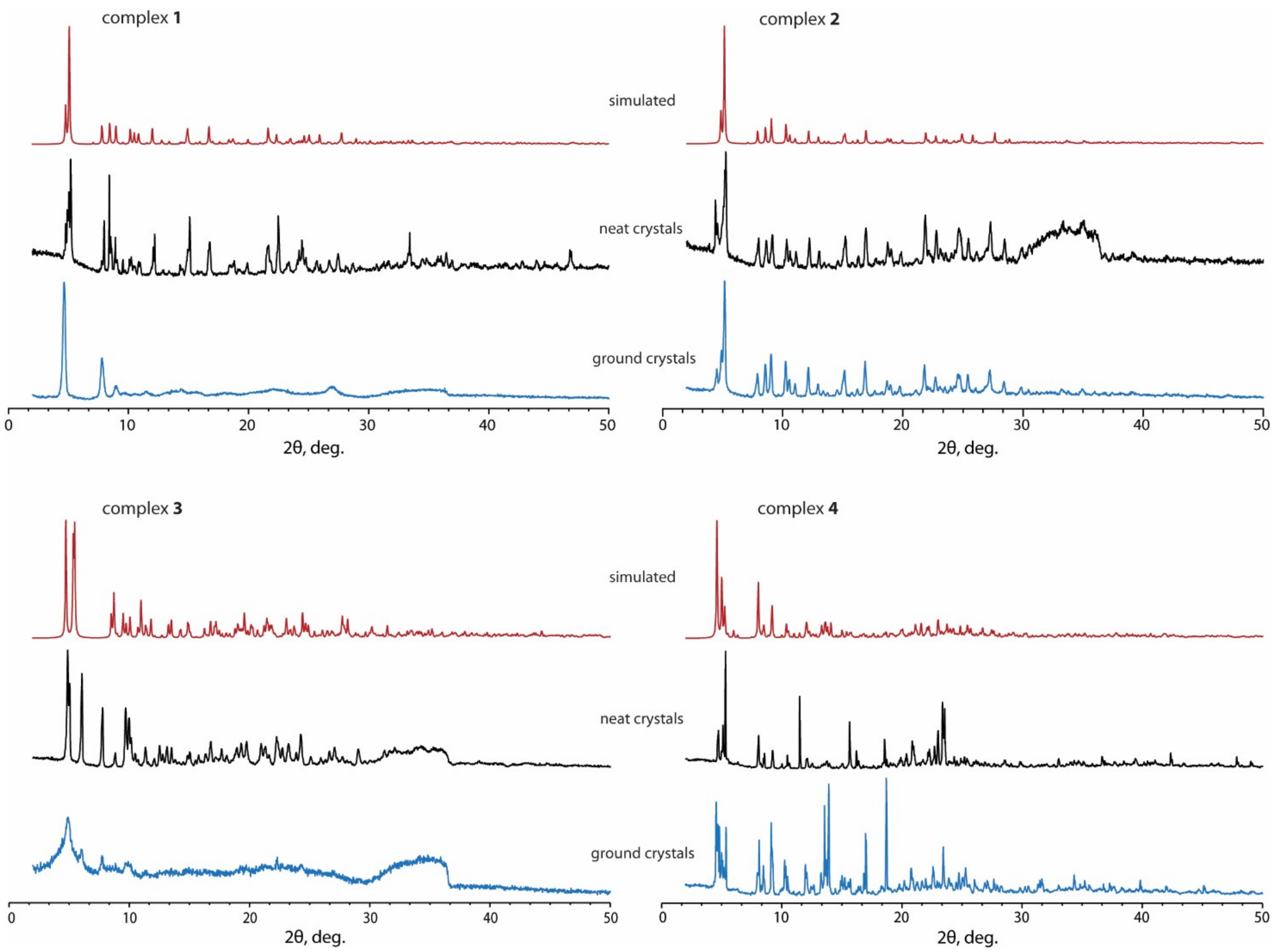

Fig. S3. Simulated and experimental powder XRD patterns for crystalline and ground samples of **1** to **4** (the region of 2θ between 30–36° shows an increased background, at high voltages and low intensity of diffraction the energy resolution of the scintillation counter is insufficient to discriminate this).

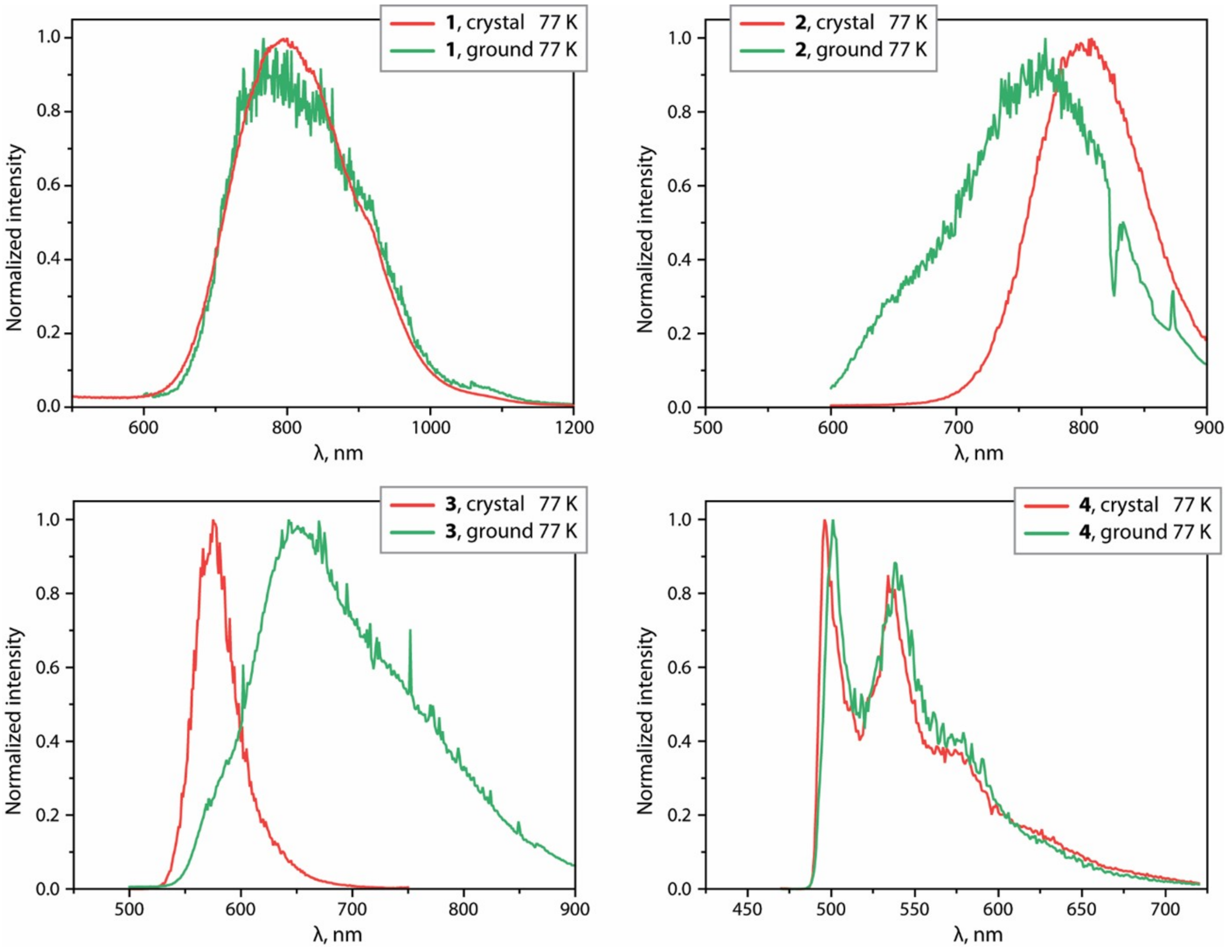

Fig. S4. Normalized photoluminescence spectra of compounds **1** to **4** in the solid state at 77 K (λ_{exc} = 375 nm, solvent-free vacuum dried solids).

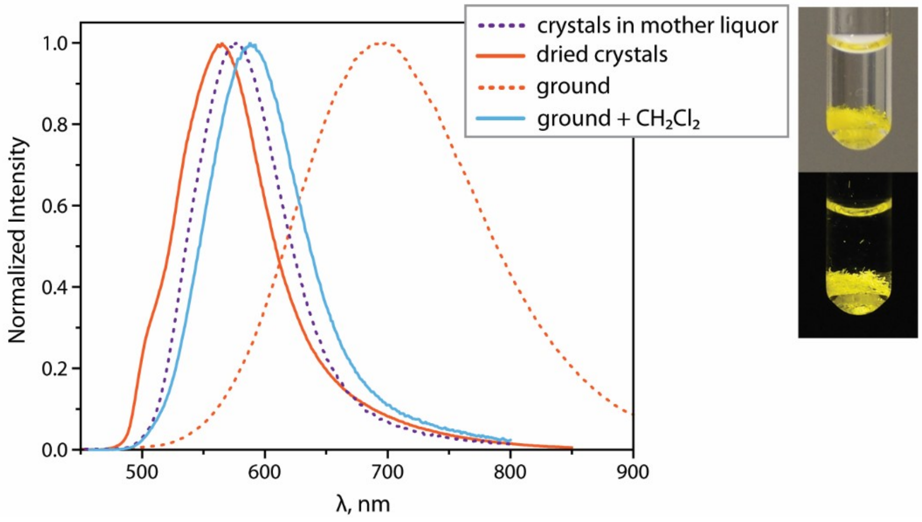

Fig. S5. Normalized photoluminescence spectra of complex **3**: solvated crystals **3**·CH_2Cl_2, dried solvent-free crystals, ground material, and treated with a drop of CH_2Cl_2 after grinding at 298 K (λ_{exc}

= 375 nm; the inset photos show the appearance of crystalline **3**·CH_2Cl_2 preserved in mother liquor under ambient and 365 nm UV light.

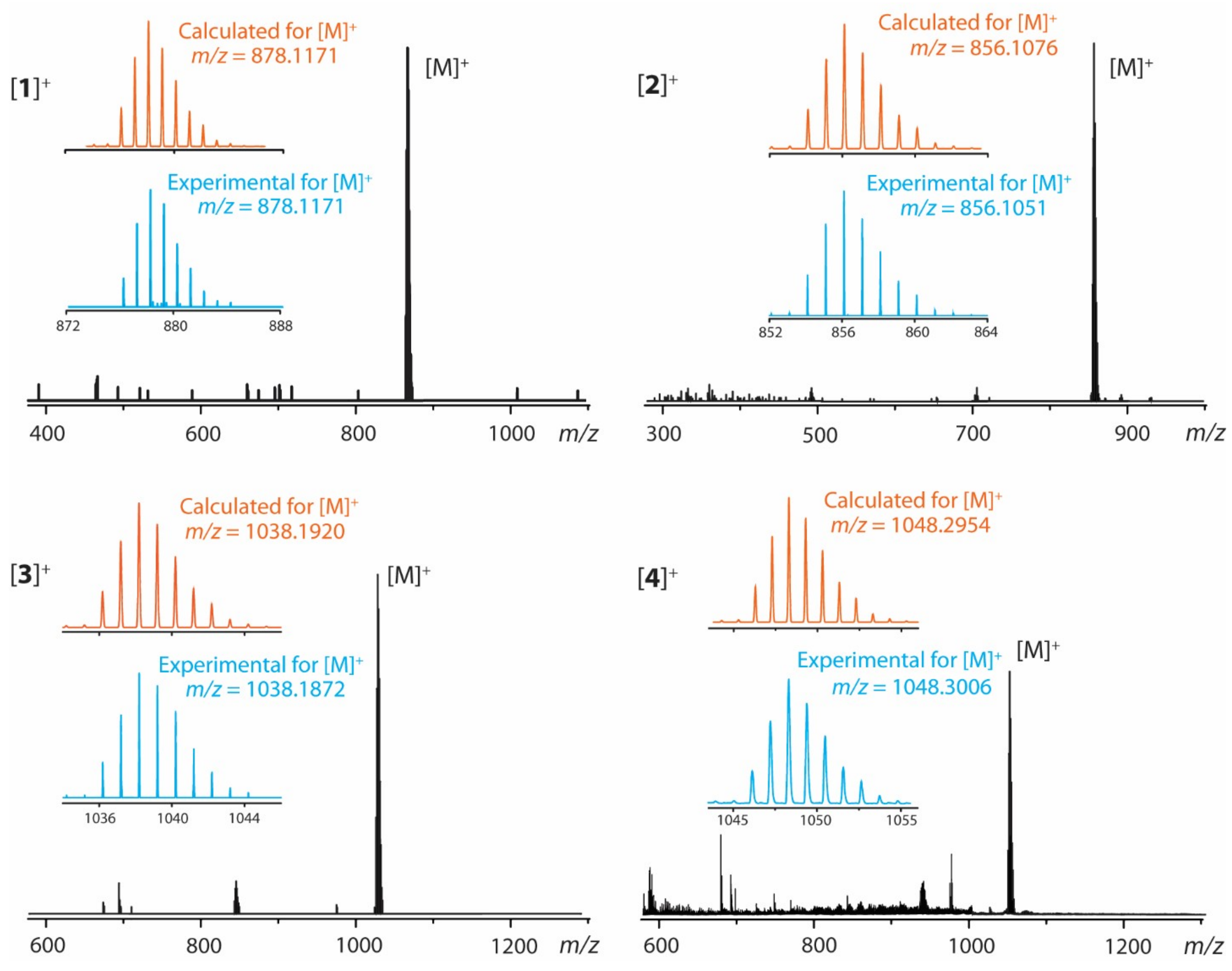

Fig. S6. ESI-MS of compounds **1** to **4**.

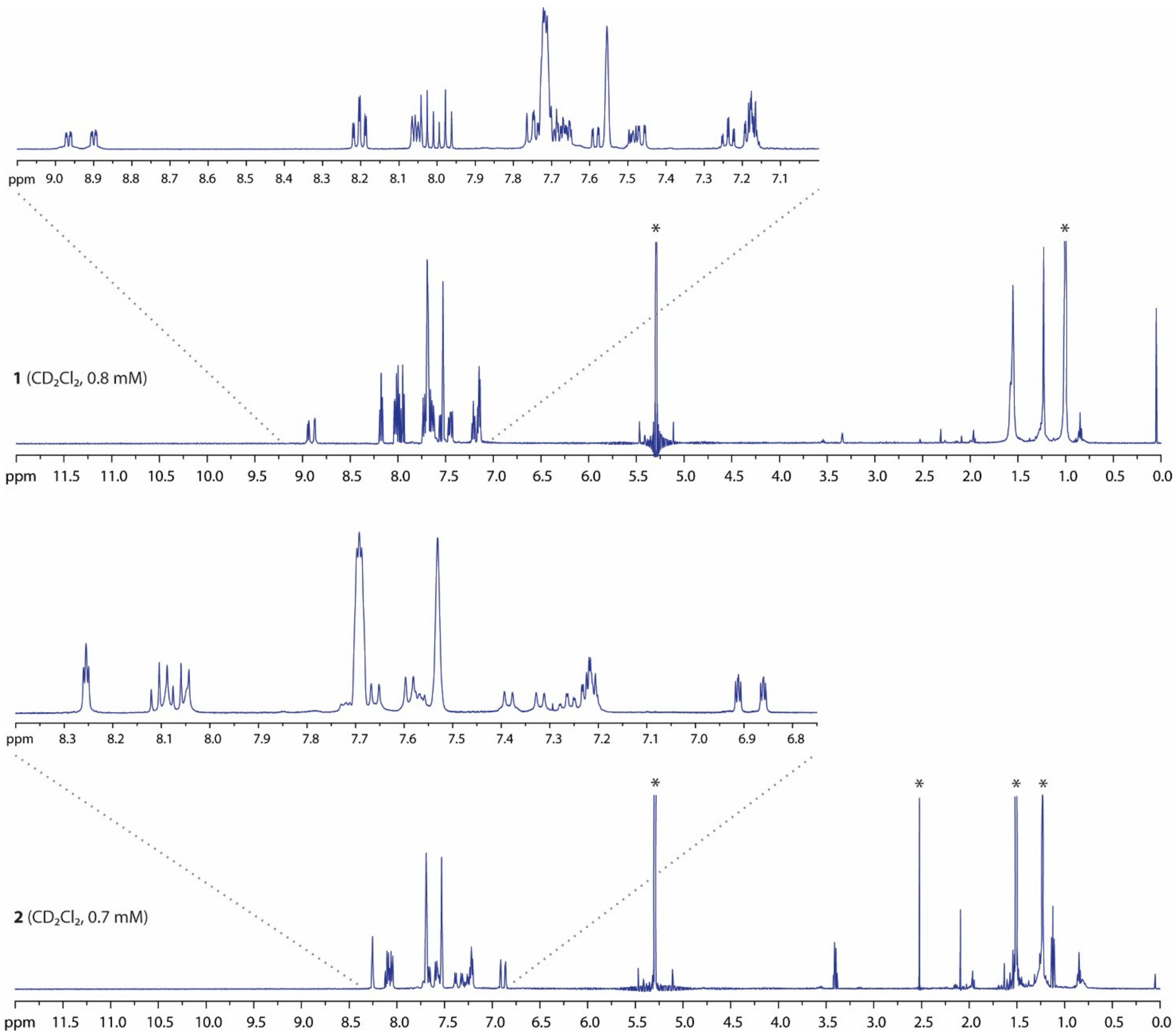

Fig. S7. 500 MHz ^{1}H NMR spectra of compounds **1** and **2** (CD_2Cl_2, 293 K; signals of high intensity from residual solvent/admixture have been cut and indicated with asterisks).

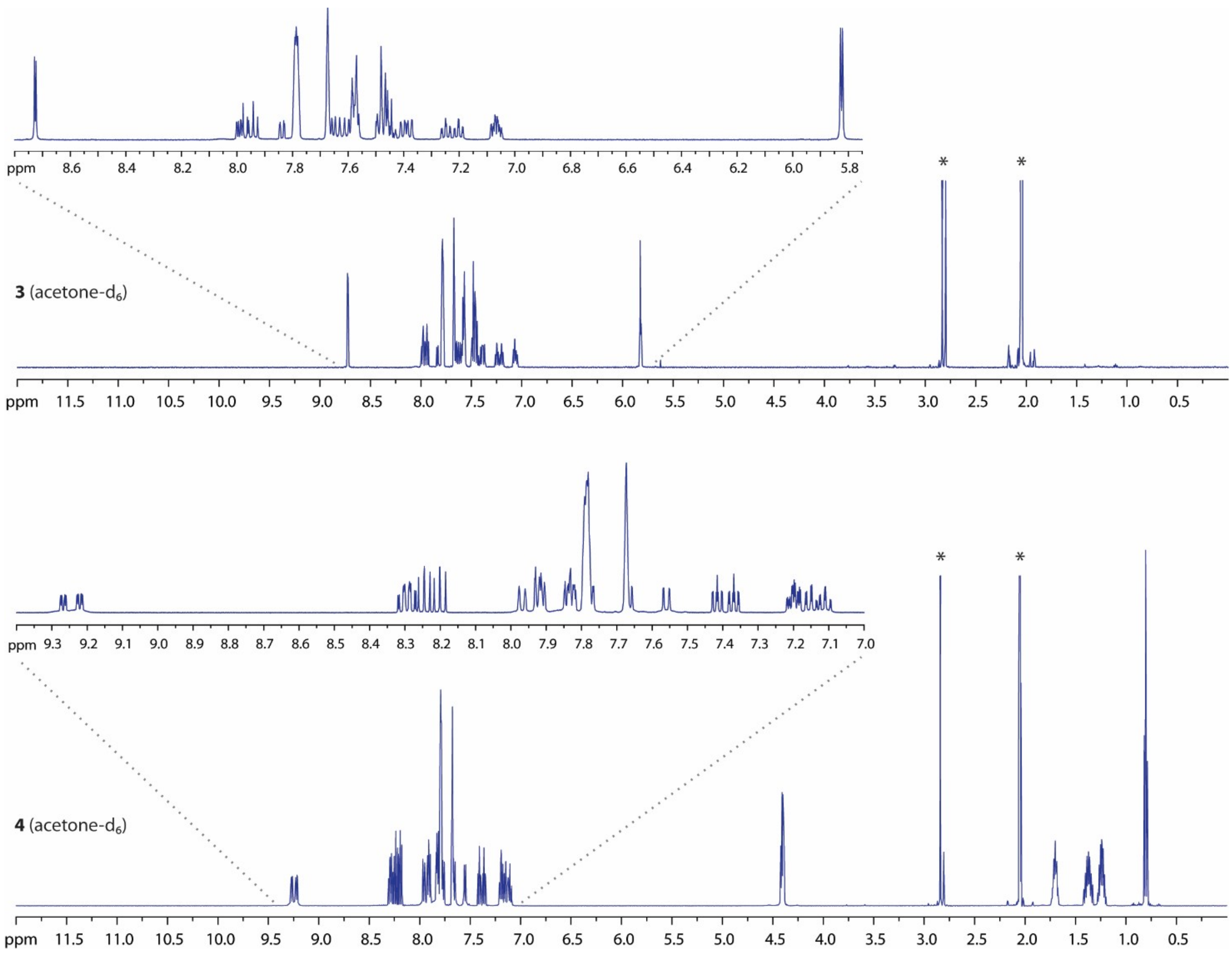

Fig. S8. 500 MHz ^{1}H NMR spectra of compounds **3** and **4** (acetone-d_6, 293 K; signals of high intensity from residual solvent/admixture have been cut and indicated with asterisks).

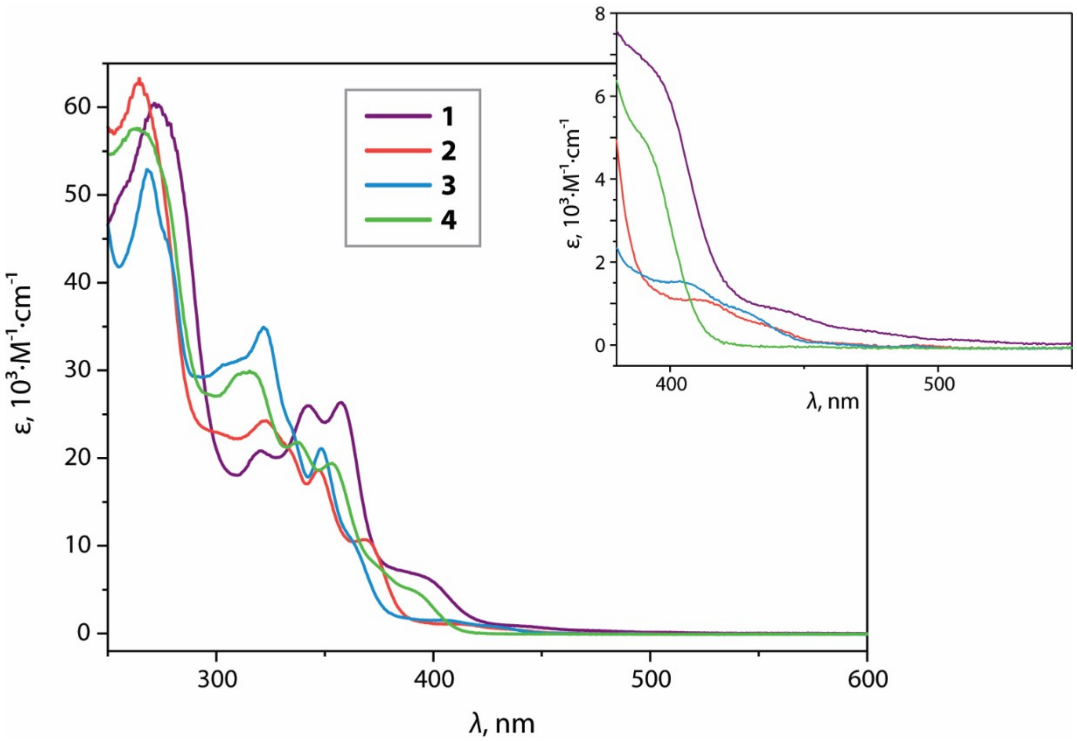

Fig. S9. UV-vis absorption spectra of compounds **1** to **4** in CH_2Cl_2 at 298 K.

Table S3. UV-vis absorption maxima of compounds **1** to **4**.[a]

Compound	λ_{abs}, nm (ε, 10^{-3} M^{-1} cm^{-1})	ΔE (eV)[b]
**1**	272 (60.5), 320 (20.8), 342 (25.9), 358 (26.3), 392sh (6.3), 443 (0.8), 470sh (0.4), 523sh (0.2)	2.37
**2**	265 (62.8), 323 (24.3), 347 (18.6), 368 (10.7), 412 (1.3), 420sh (0.8)	2.95
**3**	268 (53.0), 322 (34.9), 349 (21.0), 405 (1.6), 417sh (1.0)	2.97
**4**	263 (57.7), 315 (30.0), 337 (21.8), 353 (19.4), 390sh (5.0)	3.18

[a] Measured in CH_2Cl_2; [b] optical HOMO-LUMO gap calculated from the long-wavelength absorption bands at lowest energy.

Table S4. TD-DFT-calculated lowest-lying singlet-singlet vertical excitations and lowest singlet-triplet vertical excitations for diplatinum cations of **1** to **4** in CH_2Cl_2.

Complex	Transition	*E*, eV	λ, nm	f^a	Contributions[b]	Assignment
**1**	$S_0 \rightarrow S_1$	3.06	405	0.0010	59% H→L+1. 30% H→L	MLCT/IL
	$S_0 \rightarrow S_2$	3.09	402	0.0003	73% H-1→L, 17% H-1→L+1	MLCT/IL
	$S_0 \rightarrow S_3$	3.63	342	0.0305	31% H-6→L+1, 12% H-6→L	MLCT
	$S_0 \rightarrow S_4$	3.65	340	0.1553	40% H-2→L, 26% H-2→L+1	MLCT/LLCT
	$S_0 \rightarrow S_5$	3.72	333	0.0286	52% H-7→L, 15% H-7→L+1, 13% H-6→L	MLCT
	$S_0 \rightarrow T_1$	2.70	460	-	45% H→L+1, 22% H→L, 16% H→L+3	MLCT/IL
	$S_0 \rightarrow T_2$	2.71	458	-	56% H-1→L, 14% H-1→L+2, 13% H-1→L+1	MLCT/IL
**2**	$S_0 \rightarrow S_1$	3.29	377	0.0156	93% H→L+1	MLCT/IL
	$S_0 \rightarrow S_2$	3.31	374	0.0196	94% H-1→L	MLCT/IL
	$S_0 \rightarrow S_3$	3.93	315	0.0320	53% H-6→L+1, 35.7% H-5→L+1	MLCT
	$S_0 \rightarrow S_4$	3.98	311	0.0150	43% H→L+3, 30% H-4→L+1, 15% H-2→L+1	MLCT/LLCT
	$S_0 \rightarrow S_5$	4.01	309	0.0418	80% H-7→L, 7.32% H-4→L	MLCT
	$S_0 \rightarrow T_1$	2.78	446	-	56% H→L+1, 15% H→L+3	MLCT/IL
	$S_0 \rightarrow T_2$	2.79	444	-	58% H-1→L, 15% H-1→L+2	MLCT/IL
**3**	$S_0 \rightarrow S_1$	3.25	382	0.0112	64% H→L+1, 30% H→L	MLCT/IL
	$S_0 \rightarrow S_2$	3.27	380	0.0132	76% H-1→L, 19% H-1→L+1	MLCT/IL
	$S_0 \rightarrow S_3$	3.87	321	0.0286	32% H-6→L+1, 25% H-5→L+1, 14% H-5→L, 12% H-6→L	MLCT
	$S_0 \rightarrow S_4$	3.91	317	0.1607	39% H-2→L, 18% H-2→L+1	MLCT/LLCT
	$S_0 \rightarrow S_5$	3.95	314	0.0881	55% H-7→L, 11% H-7→L+1	MLCT
	$S_0 \rightarrow T_1$	2.77	447	-	40% H→L+1, 19% H→L+3, 17% H→L	MLCT/IL
	$S_0 \rightarrow T_2$	2.78	445	-	48% H-1→L, 17% H-1→L+2, 13% H-1→L+1	MLCT/IL
**4**	$S_0 \rightarrow S_1$	3.51	353	0.0914	52% H→L+1, 41% H→L	MLCT/IL
	$S_0 \rightarrow S_2$	3.53	351	0.0984	68% H-1→L, 24% H-1→L+1	MLCT/IL
	$S_0 \rightarrow S_3$	3.92	317	0.4409	49% H-2→L, 19% H-2→L+1, 14% H-3→L+1	MLCT/IL
	$S_0 \rightarrow S_4$	3.96	313	0.0731	54% H-3→L, 25% H-2→L+1	MLCT/IL
	$S_0 \rightarrow S_5$	4.15	299	0.3347	31% H-4→L, 20% H-5→L+1, 17% H-5→L, 10% H-4→L+1	MLCT/IL
	$S_0 \rightarrow T_1$	2.81	441	-	41% H→L+1, 25% H→L	MLCT/IL
	$S_0 \rightarrow T_2$	2.82	440	-	47% H-1→L, 22% H-1→L+1	MLCT/IL

[a] Oscillator strength; [b] L = LUMO, H = HOMO, rounded values.

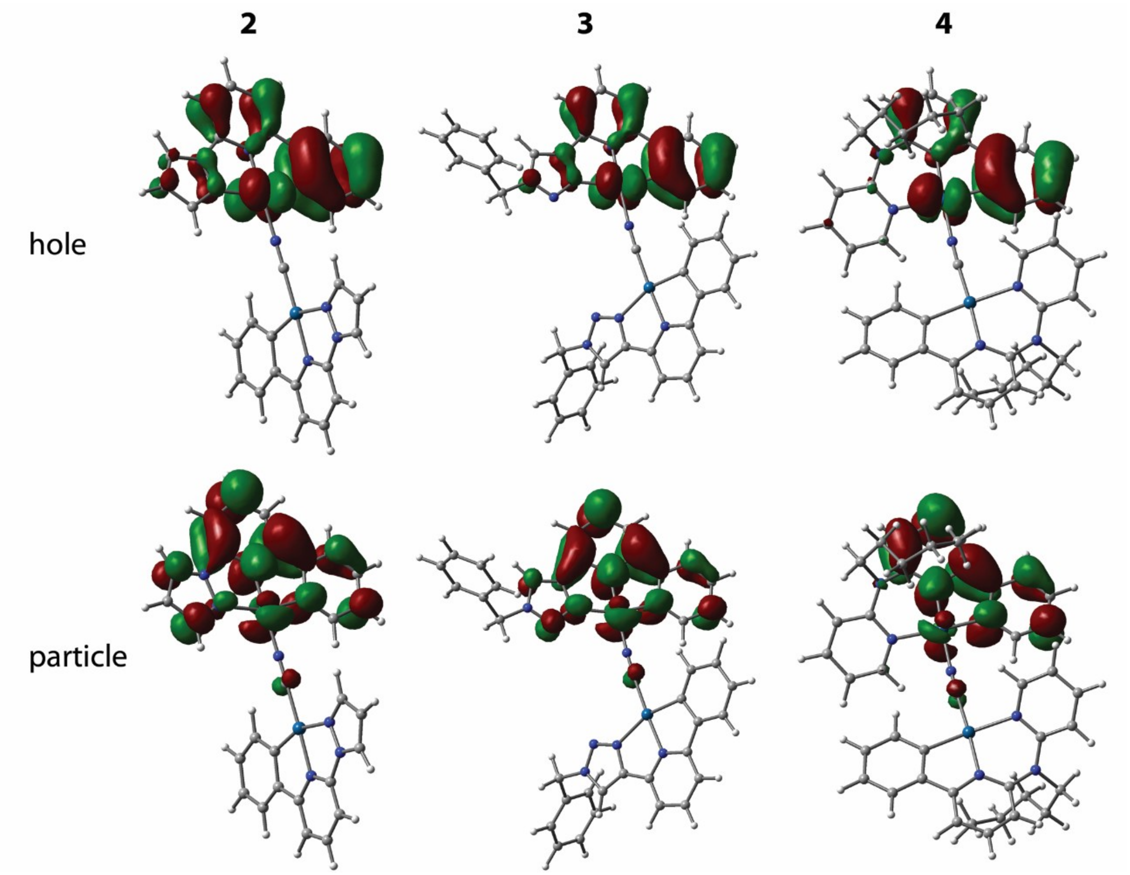

Fig. S10. TD-DFT-calculated natural transition orbital hole-particle pair for the vertical $S_0 \rightarrow S_1$ transitions for the diplatinum cations of **2** to **4** in CH_2Cl_2. For **1**, see ref. [16].

Table S5. Photophysical data of mononuclear congeners [Pt(C^N^/*N)(CN)] (**1_mono** to **4_mono**) in CH_2Cl_2 solution.

Complex	$\lambda_{em,max}$		τ_{av}, µs [a]		Φ_L (±0.02/0.05)		k_r, 10^5 s^{-1}	k_{nr}, 10^5 s^{-1}
	298 K	77 K	298 K[b]	77 K	298 K[b]	77 K	298 K	
**1_mono**[c]	562	511, 550, 690	2.199±0.016	2.8 (710 nm), 3.0 (690 nm), 13.7 (510 nm)	0.1	0.86 (abs. 0.03)	0.45±0.09	4.1±0.1
**2_mono**[d]	502, 537, 575sh	488, 525, 625	10.91±0.02	18.6 (490 nm), 5.1 (625 nm)	0.49	0.95	0.45±0.02	0.47±0.02
**3_mono**[d]	505, 538, 576sh	495,533, 590, 606	11.214±0.018	10.5 (495 nm), 5.2 (600 nm)	0.43	0.95	0.38±0.02	0.51±0.02
**4_mono**	492, 528 560sh	490, 528, 560, 608sh	9.78±0.07	49.0	0.33	0.95	0.34±0.02	0.69±0.03

[a] Amplitude-weighted average lifetimes determined by the equation $\tau_{av} = \Sigma A_i \tau_i$, A_i = weight of the i-th component; [b] under an inert atmosphere of Ar; [c] data from ref. [16]; [d] the synthesis and solid-state luminescence were reported in ref. [17].

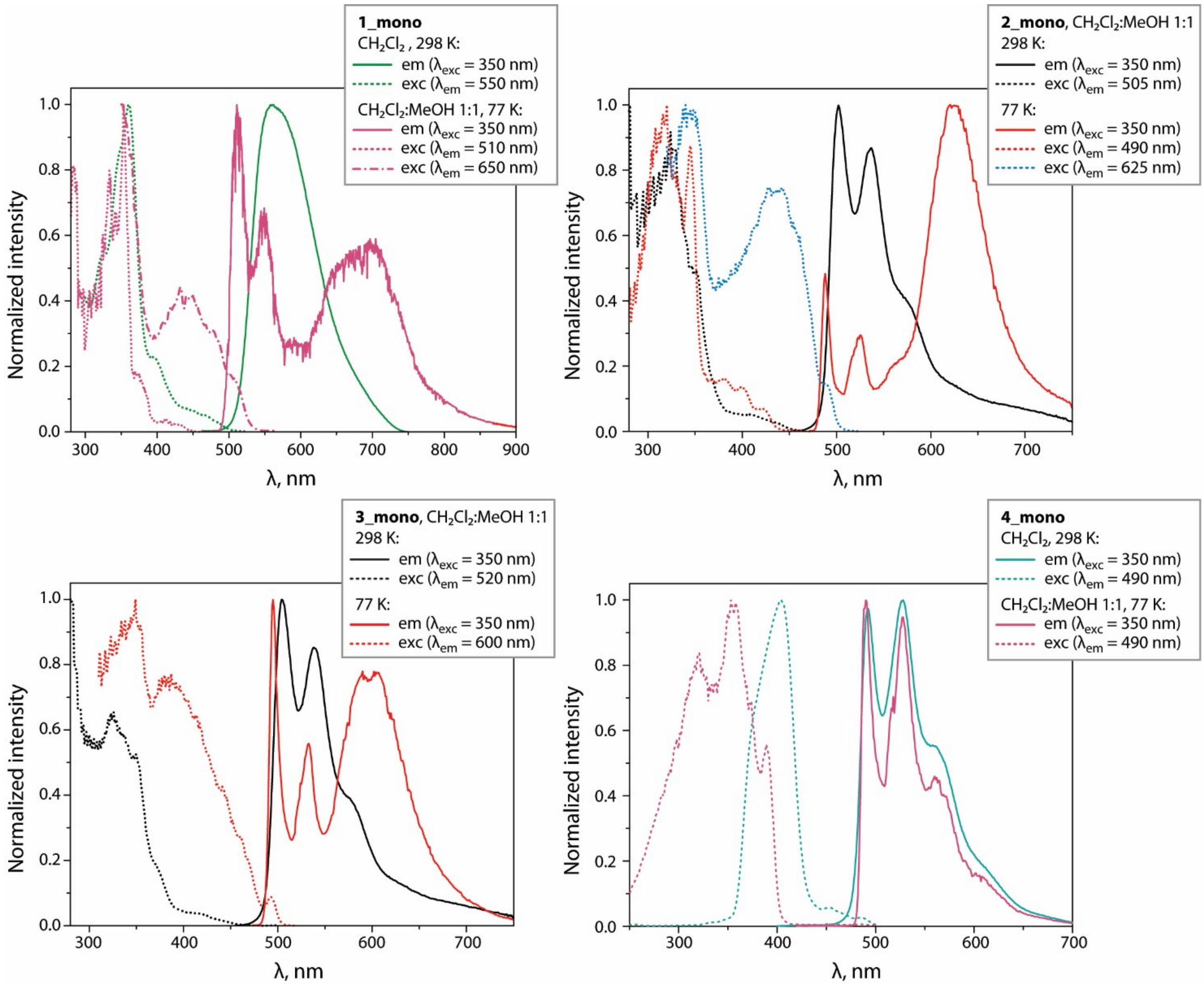

Fig. S11. Normalized excitation and emission spectra of complexes [Pt(C^N^/*N)(CN)] (**1_mono** to **4_mono**) in solution at 298 and 77 K.

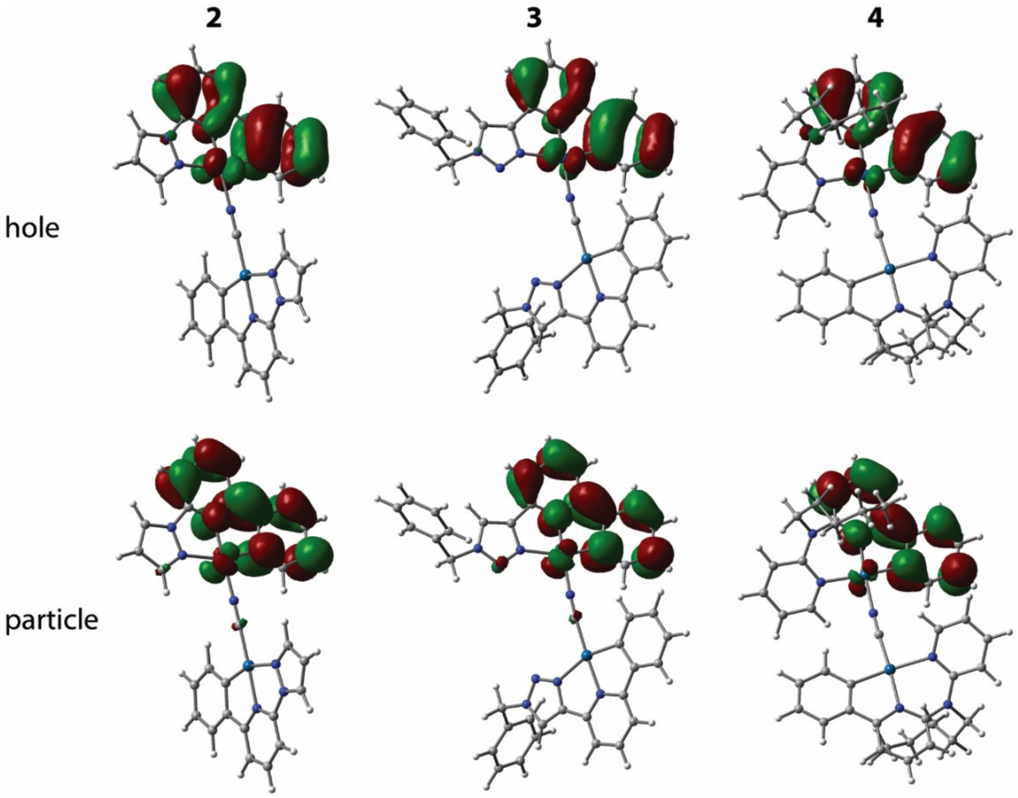

Fig. S12. Natural transition orbital hole-particle pair for the vertical $T_1 \rightarrow S_0$ transitions for the diplatinum cations of **2** to **4** in CH_2Cl_2 at the optimized excited state geometries.

Table S6. DFT-predicted vibrationally resolved phosphorescence for complexes **1** to **4** and vertical phosphorescence for their aggregates.

Complex	λ, nm	Assignment
**1**[a]	544, 596, 655	MLCT/IL
**2**[a]	530, 577, 618, 629	IL/MLCT
**3**[a]	528, 576, 618, 630	IL/MLCT
**4**[a]	541, 588, 642	IL/MLCT
**1 dimer A (XRD)**[b]	785	MMLCT
**1 dimer B (XRD)**[b]	698	MMLCT
**2 dimer A (XRD)**[b]	671	MMLCT
**3 dimer (XRD)**[b]	584	MMLCT
**1 dimer (free)**[c]	661	MMLCT
**1 trimer (free)**[c]	782	MMLCT
**2 dimer B (free)**[c]	573	MMLCT
**2 trimer(free)**[c]	685	MMLCT

[a] Vibrationally resolved phosphorescence, 298 K, CH_2Cl_2; [b] optimized geometry based on crystallographic data, vertical phosphorescence in vacuum (see Figs. S14, S15); [c] optimized free geometry, vertical phosphorescence in vacuum (see Figs. S14, S15).

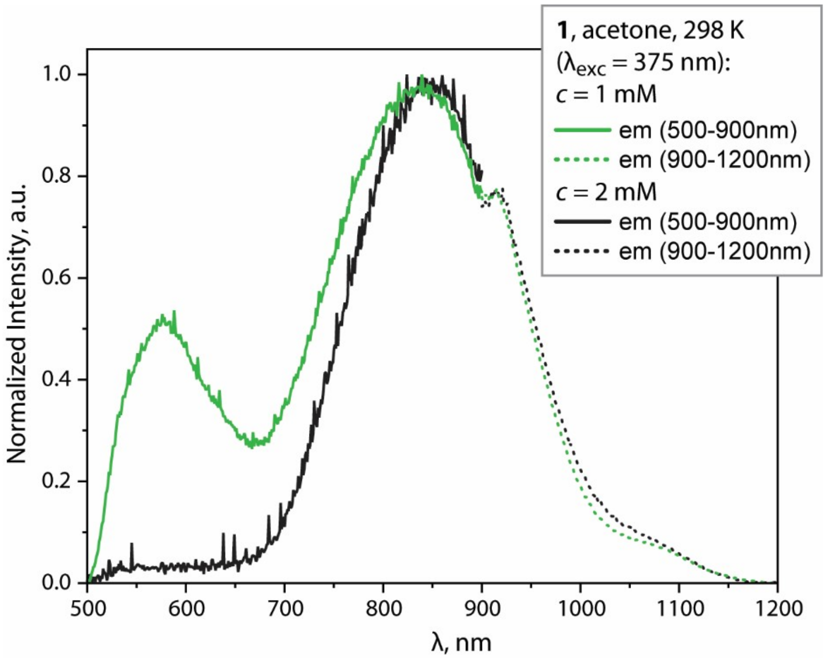

Fig. S13. Normalized room temperature emission spectra of **1** in acetone at $c = 1$ mM (green line) and 2 mM (black line).

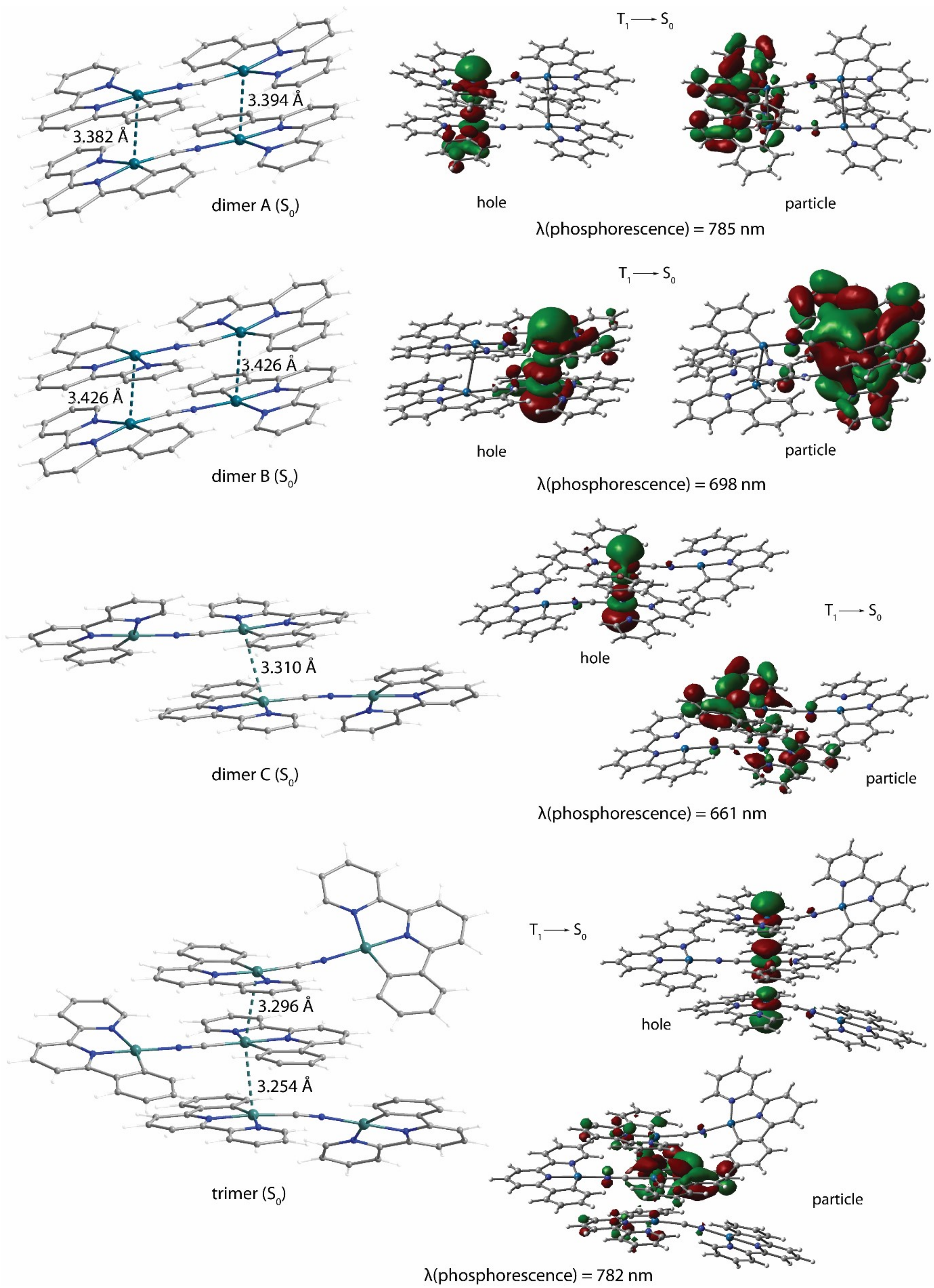

Fig. S14. Left: DFT-optimized ground state geometries of the dimers $[\mathbf{1}]_2^{2+}$ (A and B are based on crystallographic data, C has head-to-tail orientation of cyclometalated fragments) and the trimer $[\mathbf{1}]_3^{3+}$. Right: natural transition orbital hole-particle pairs for the $T_1 \rightarrow S_0$ transitions.

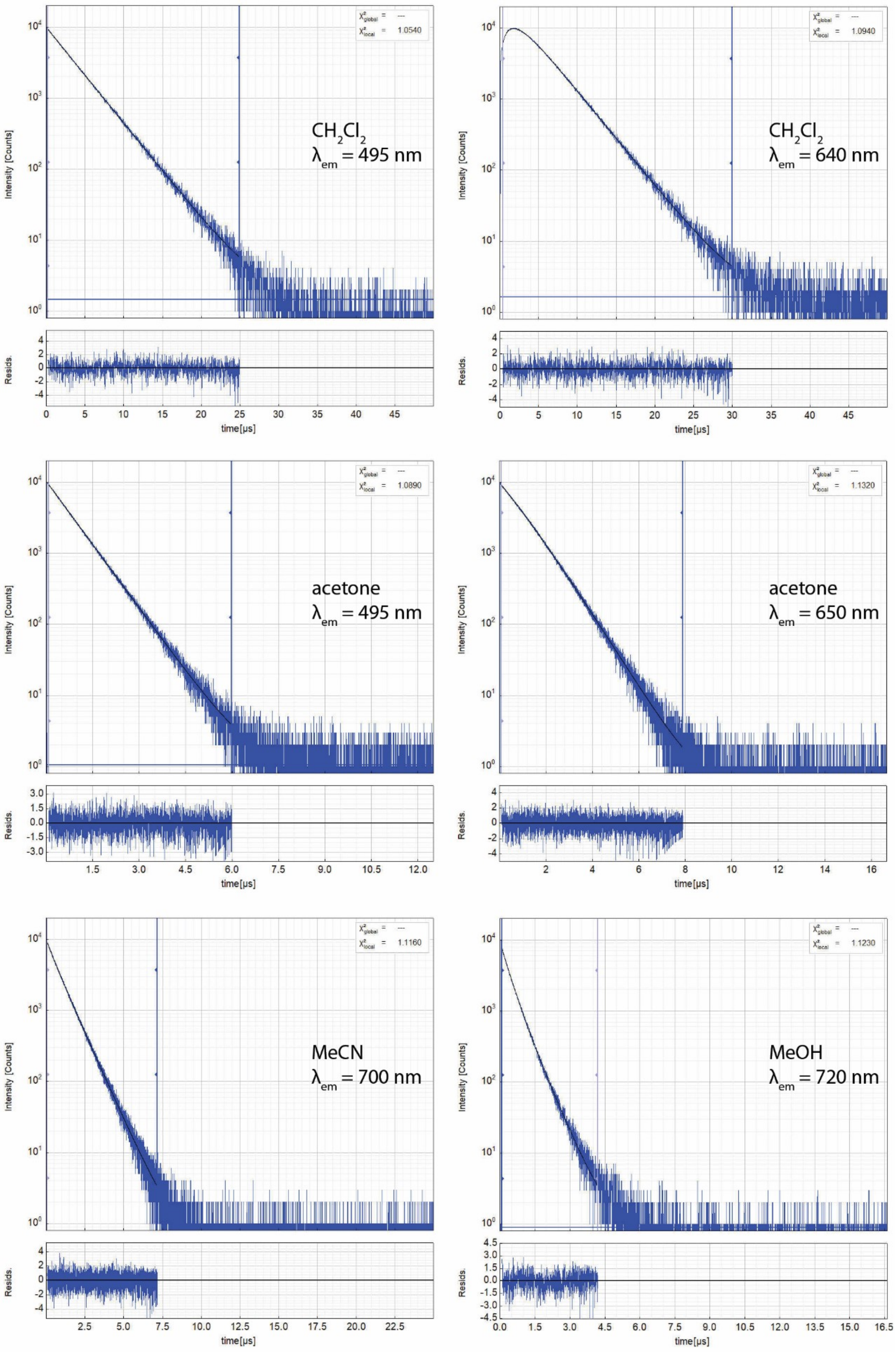

Fig. S15. Time-resolved photoluminescence decay profiles for **2** in CH_2Cl_2, acetone, MeCN, MeOH (c = 1 mM, room temperature) monitored at different emission wavelengths.

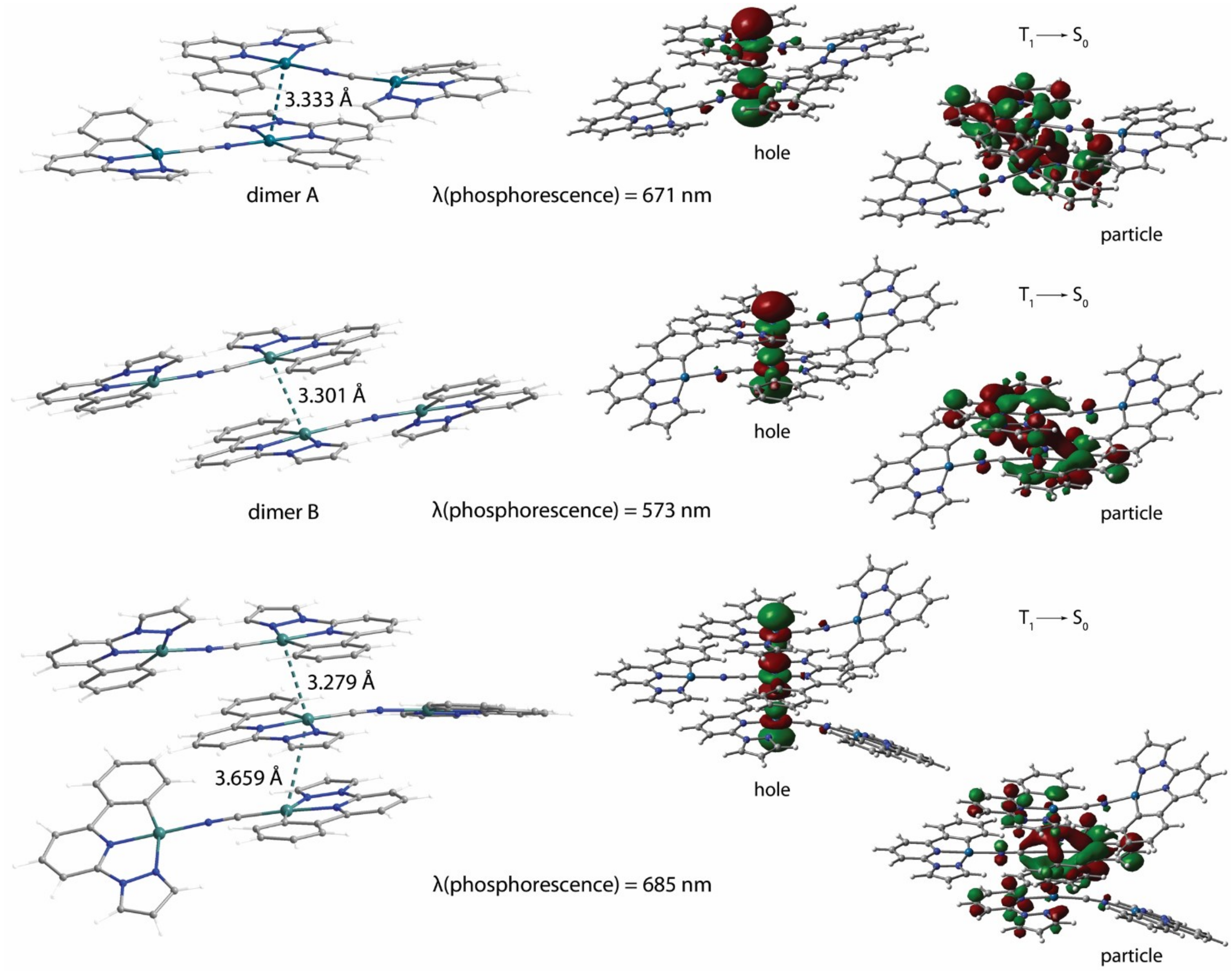

Fig. S16. Left: optimized ground state geometries of the dimers $[\mathbf{2}]_2^{2+}$ (A is based on crystallographic data, B has a head-to-tail orientation of the cyclometalated fragments) and the trimer $[\mathbf{2}]_3^{3+}$. Right: natural transition orbital hole-particle pairs for the $T_1 \rightarrow S_0$ transitions.

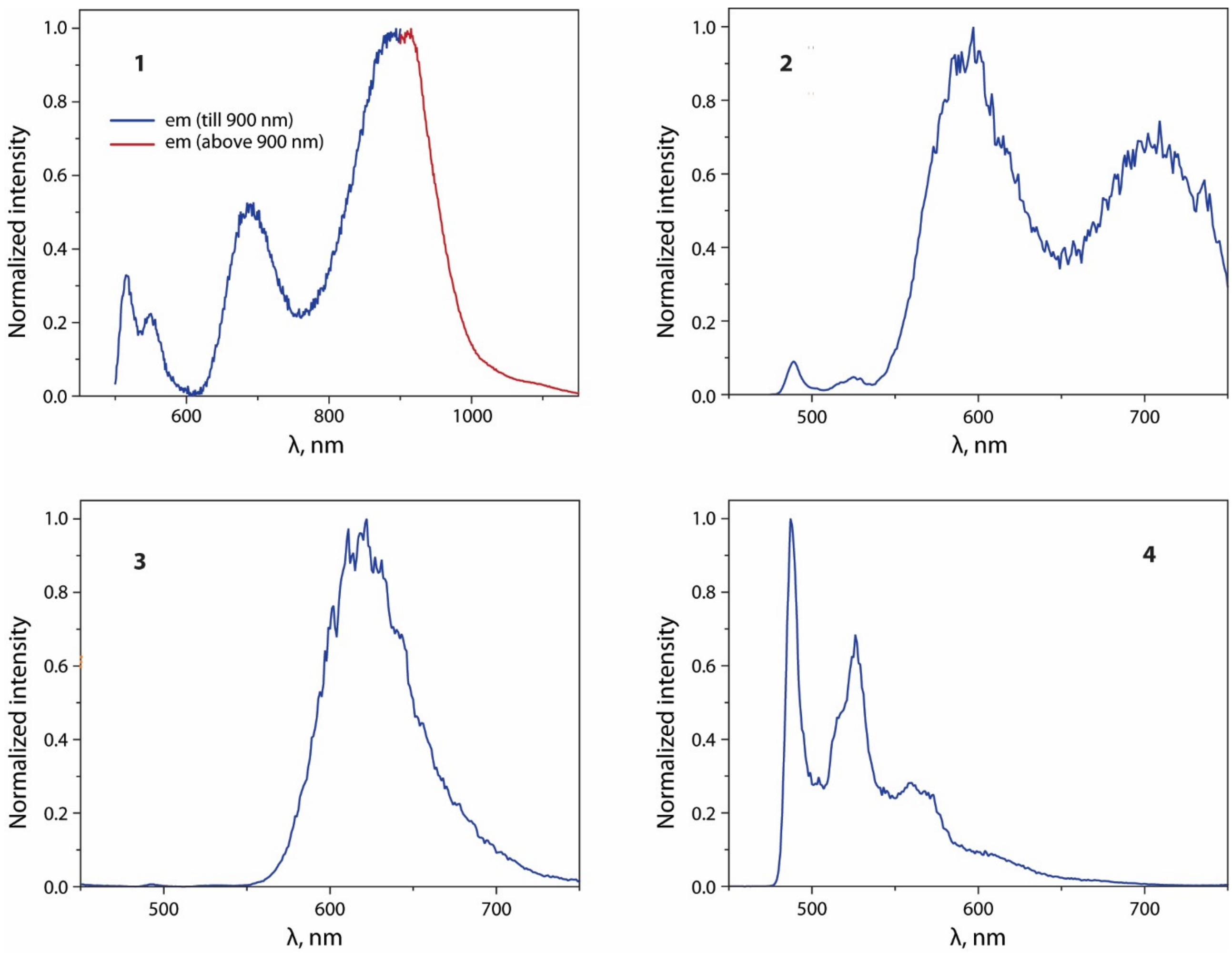

Fig. S17. Normalized emission spectra of optically dilute **1** to **4** in frozen (77 K) CH_2Cl_2:MeOH 1:1 v/v mixture, λ_{exc} = 350 nm.

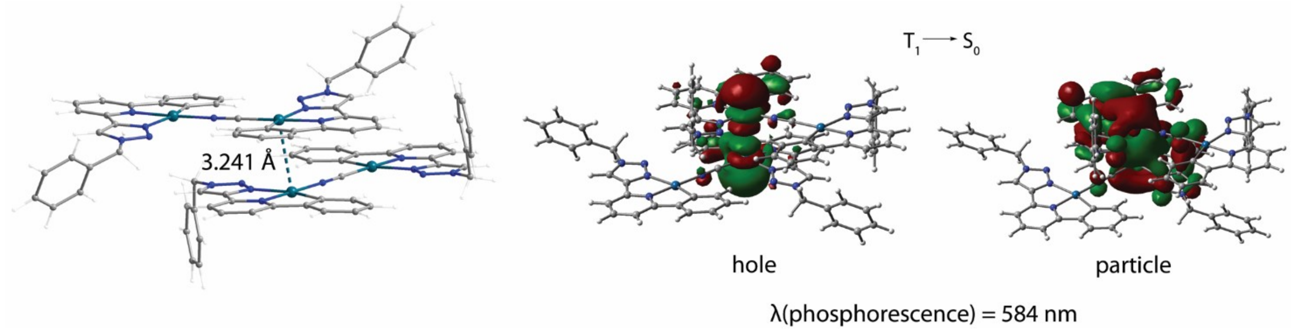

Fig. S18. Left: DFT-optimized ground state geometry of the dimer [**3**]$_2^{2+}$ based on crystallographic data. Right: natural transition orbital hole-particle pairs for the $T_1 \rightarrow S_0$ transition.

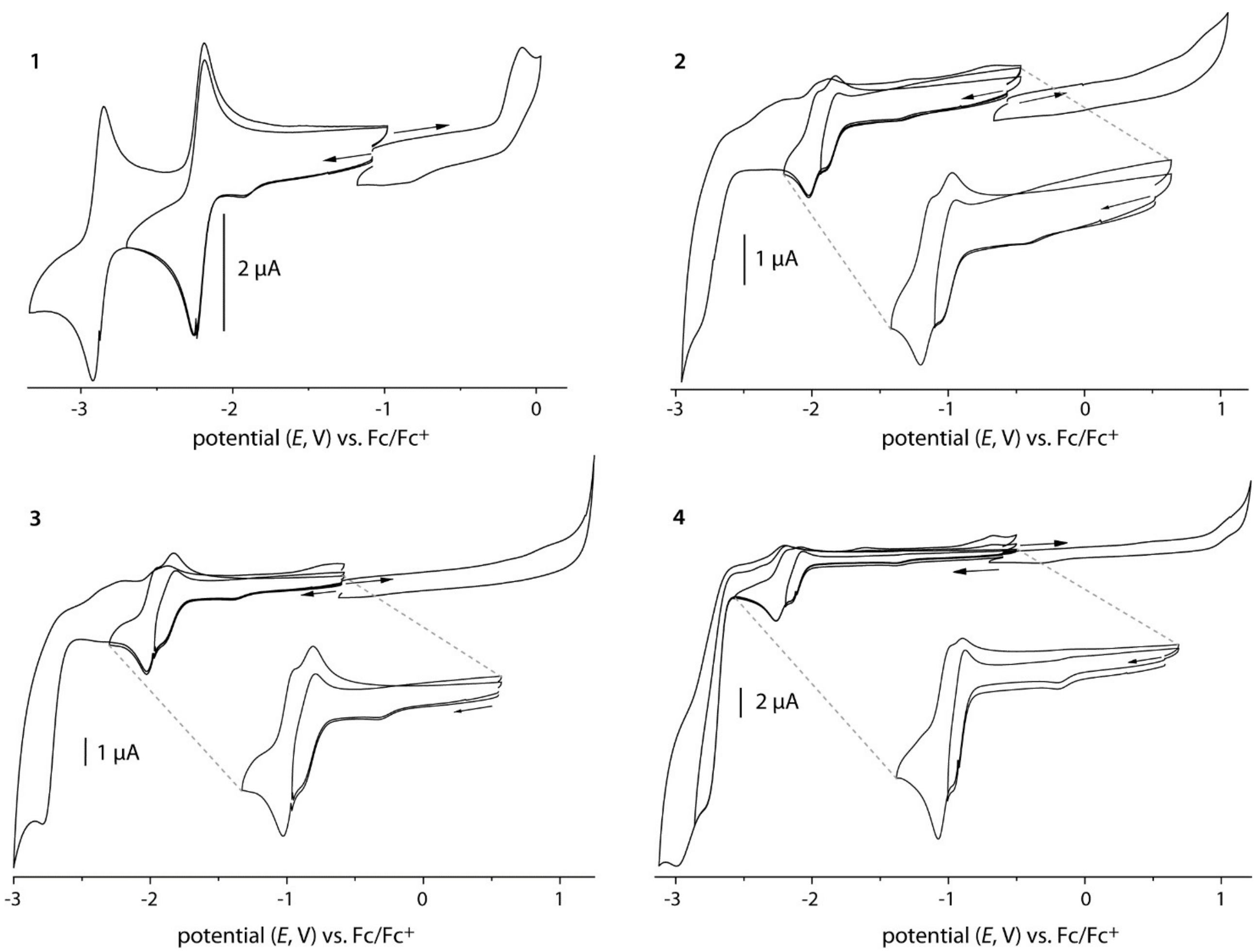

Fig. S19. Cyclic voltammograms of **1** to **4** in *n*-Bu_4NPF_6/DMF at a scan rate of 100 mV/s at 298 K.

Table S7. Redox potentials of the Pt(II) complexes **1** to **4**.[a]

Complex	E_{pc} Red4	E_{pc} Red3	$E_{1/2}$ Red2	$E_{1/2}$ Red1	E_{pa} Ox1	Δ*E* Red1-Red2	Δ*E* Ox1-Red1
**1**	-	-	−2.88	−2.21	−0.09	0.67	2.12
**2**	-	−2.82	−1.98	−1.85	0.87	0.13	2.72
**3**	-	−2.79	−1.97	−1.86	0.89	0.11	2.75
**4**	−2.99	−2.79	−2.21	−2.11	1.05	0.10	3.16

[a] From cyclic voltammetry in *n*-Bu_4NPF_6/DMF. Potentials in V *vs.* ferrocene/ferrocenium, half-wave potentials $E_{1/2}$ for (partially) reversible processes, cathodic peak potentials E_{pc} and anodic peak potentials E_{pa} for irreversible reductions; accuracy of potentials: ±0.003 V.

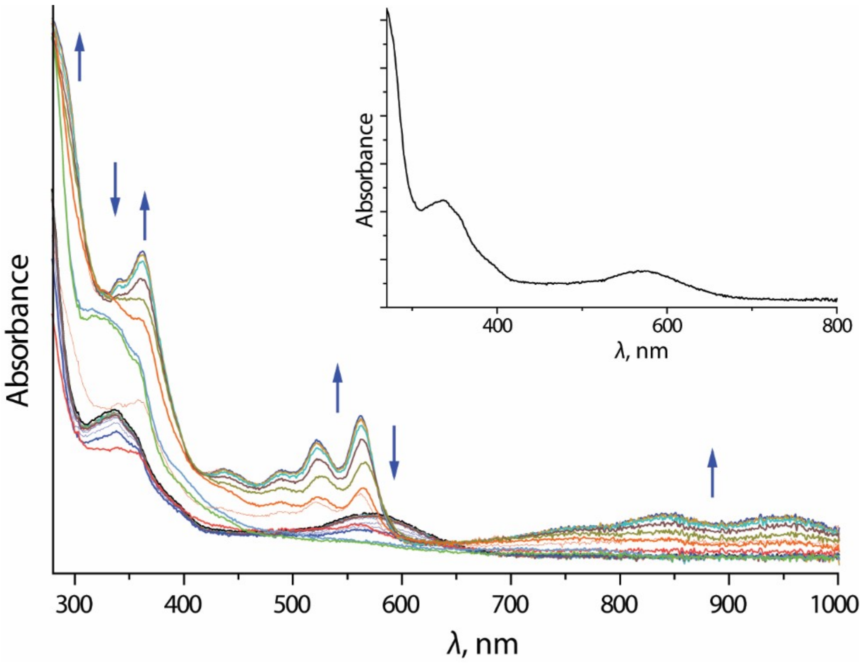

Fig. S20. UV-vis absorption spectra recorded during cathodic electrolysis (reduction) of compound **1** in *n*-Bu_4NPF_6/DMF (starting spectrum as insert).

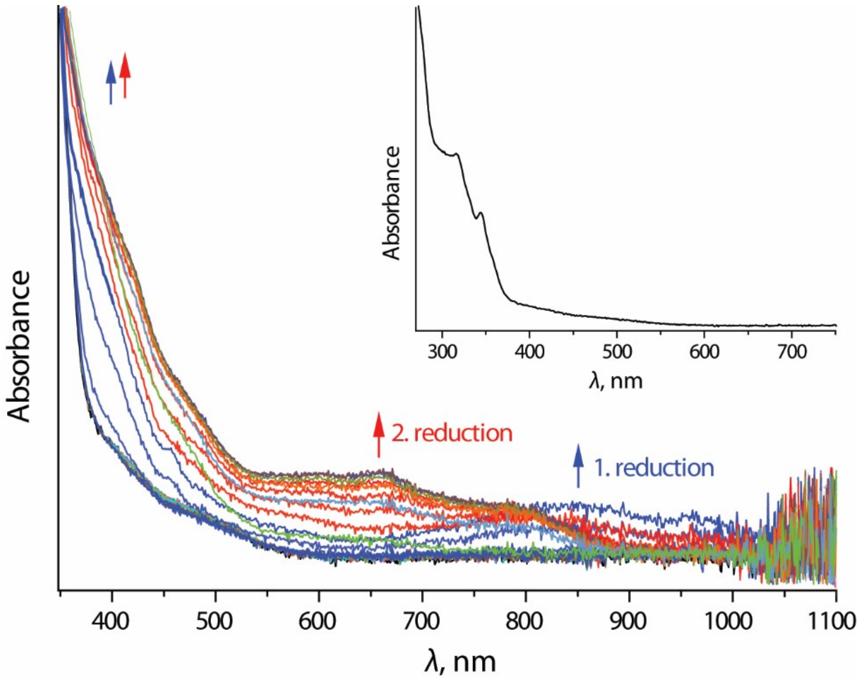

Fig. S21. UV-vis absorption spectra recorded during cathodic electrolysis (reduction) of compound **2** in *n*-Bu_4NPF_6/DMF (initial spectrum as insert).

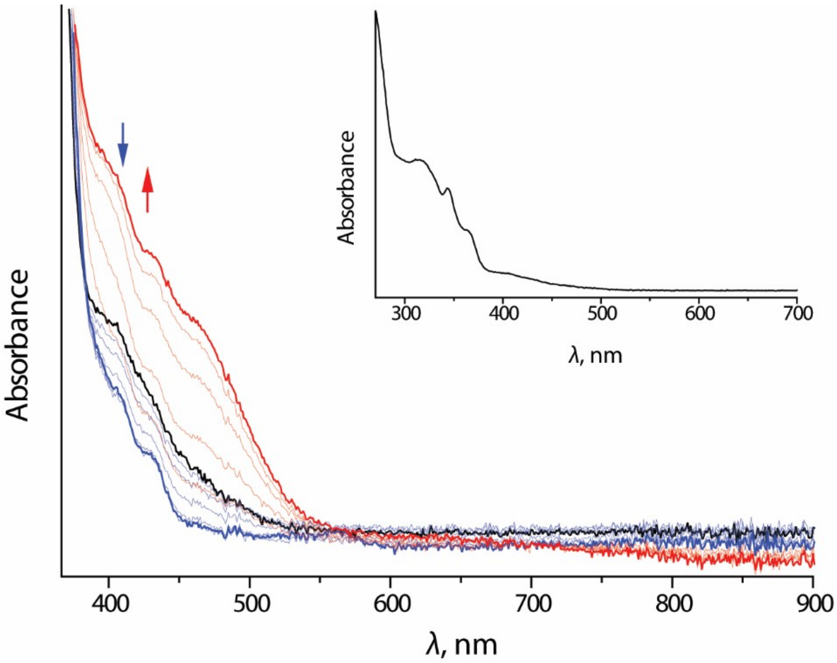

Fig. S22. UV-vis absorption spectra recorded during cathodic electrolysis (reduction) of compound **3** in *n*-Bu_4NPF_6/DMF (initial spectrum as insert).

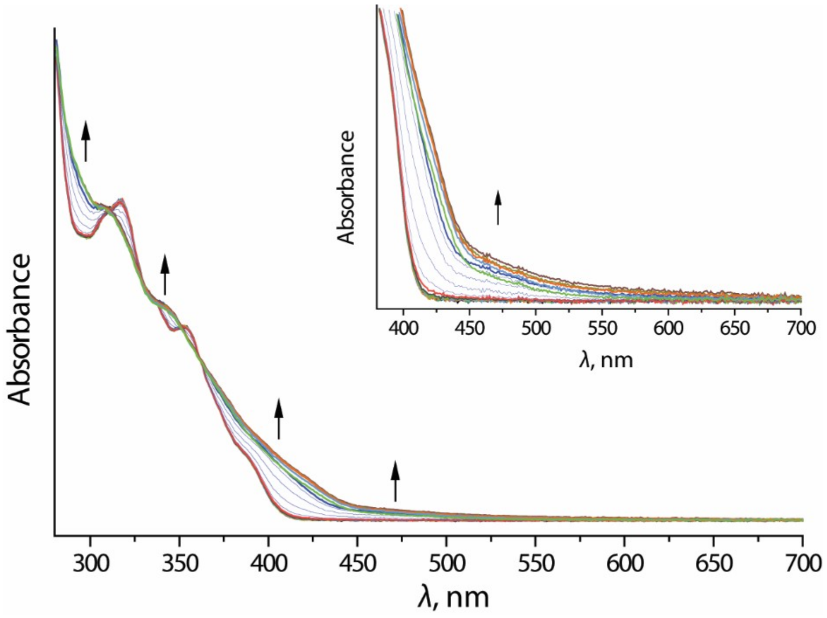

Fig. S23. UV-vis absorption spectra recorded during cathodic electrolysis (reduction) of compound **4** in *n*-Bu_4NPF_6/DMF (long-wavelength zoom as insert).

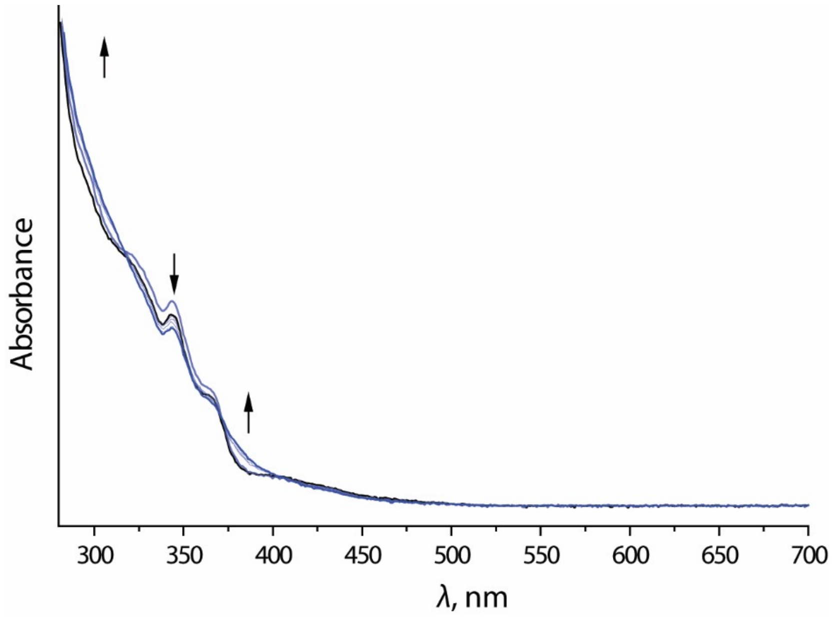

Fig. S24. UV-vis absorption spectra recorded during anodic electrolysis (oxidation) of compound **3** in *n*-Bu_4NPF_6/DMF.

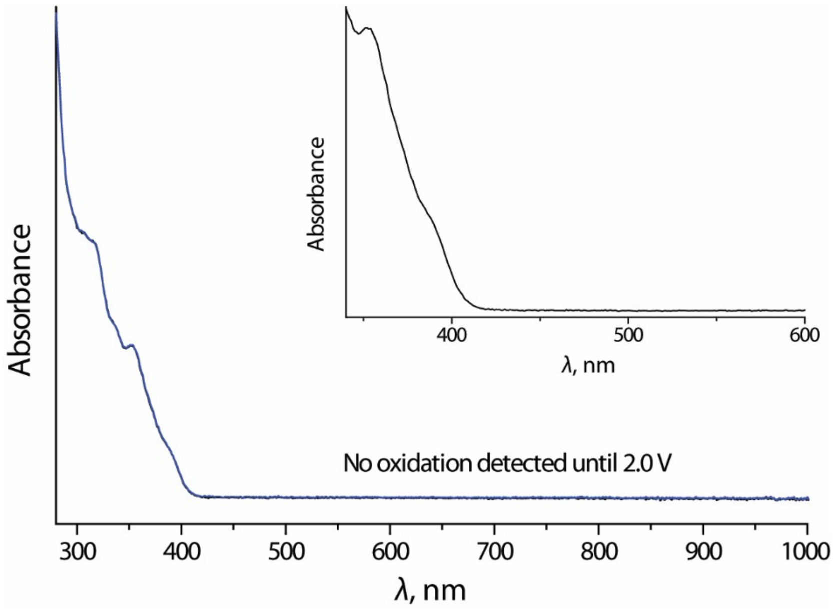

Fig. S25. UV-vis absorption spectra recorded during anodic electrolysis (oxidation) of compound **4** in *n*-Bu_4NPF_6/DMF (initial spectrum as insert).

References

1. T.-C. Cheung, K.-K. Cheung, S.-M. Peng, C.-M. Che, *J. Chem. Soc., Dalton Trans.*, 1996, , 1645–1651.
2. M. Hebenbrock, L. Stegemann, J. Kösters, N. L. Doltsinis, J. Müller, C. A. Strassert, *Dalton Trans.*, 2017, **46**, 3160–3169.
3. C. F. Harris, D. A. K. Vezzu, L. Bartolotti, P. D. Boyle, S. Huo, *Inorg. Chem.*, 2013, **52**, 11711−11722.
4. S. Buss, C. A. Strassert, to be published elsewhere.
5. (a) W. Kaim, A. Klein, eds., *Spectroelectrochemistry*, The Royal Society of Chemistry, Cambridge, UK, 2008; (b) W. Kaim, J. Fiedler, *Chem. Soc. Rev.*, 2009, **38**, 3373–3382.
6. APEX2 - Software Suite for Crystallographic Programs, Bruker AXS, Inc.,2010.
7. G. M. Sheldrick, SADABS-2008/1 – Bruker AXS Area Detector Scaling and Absorption Correction, Bruker AXS, 2008.
8. G. M. Sheldrick, *Acta Crystallogr. C: Struct. Chem.*, 2015, **71**, 3–8.
9. L. J. Farrugia, *J. Appl. Crystallogr.*, 2012, **45**, 849–854.
10. (a) S. Dapprich, I. Komáromi, K. S. Byun, K. Morokuma, M. J. Frisch, *J. Mol. Struct.: THEOCHEM*, 1999, **461–462**, 1–21; (b) T. Vreven, K. S. Byun, I. Komáromi, S. Dapprich, J. A. Montgomery, Jr., K. Morokuma, M. J. Frisch, *J. Chem. Theory Comput.*, 2006, **2**, 815–826.
11. A. K. Rappe, C. J. Casewit, K. S. Colwell, W. A. Goddard, W. M. Skiff, *J. Am. Chem. Soc.*, 1992, **114**, 10024–10035.
12. M. J. Frisch, G. W. Trucks, H. B. Schlegel, G. E. Scuseria, M. A. Robb, J. R. Cheeseman, G. Scalmani, V. Barone, G. A. Petersson, H. Nakatsuji, X. Li, M. Caricato, A. V. Marenich, J. Bloino, B. G. Janesko, R. Gomperts, B. Mennucci, H. P. Hratchian, J. V. Ortiz, A. F. Izmaylov, J. L. Sonnenberg, D. Williams-Young, F. Ding, F. Lipparini, F. Egidi, J. Goings, B. Peng, A. Petrone, T. Henderson, D. Ranasinghe, V. G. Zakrzewski, J. Gao, N. Rega, G. Zheng, W. Liang, M. Hada, M. Ehara, K. Toyota, R. Fukuda, J. Hasegawa, M. Ishida, T. Nakajima, Y. Honda, O. Kitao, H. Nakai, T. Vreven, K. Throssell, J. A. J. Montgomery, J. E. Peralta, F. Ogliaro, M. J. Bearpark, J. J. Heyd, E. N. Brothers, K. N. Kudin, V. N. Staroverov, T. A. Keith, R. Kobayashi, J. Normand, K. Raghavachari, A. P. Rendell, J. C. Burant, S. S. Iyengar, J. Tomasi, M. Cossi, J. M. Millam, M. Klene, C. Adamo, R. Cammi, J. W. Ochterski, R. L. Martin, K. Morokuma, O. Farkas, J. B. Foresman, D. J. Fox, Gaussian 16, Revision A.03, Gaussian, Inc., 2016.
13. H. S. Yu, X. He, S. L. Li, D. G. Truhlar, *Chem. Sci.*, 2016, **7**, 5032–5051.

14. (a) F. Weigend, R. Ahlrichs, *Phys. Chem. Chem. Phys.*, 2005, **7**, 3297–3305; (b) D. Andrae, U. Häußermann, M. Dolg, H. Stoll, H. Preuß, *Theor. Chem. Acc.*, 1990, **77**, 123–141.
15. (a) V. Barone, M. Cossi, *J. Phys. Chem. A*, 1998, **102**, 1995–2001; (b) M. Cossi, N. Rega, G. Scalmani, V. Barone, *J. Comput. Chem.*, 2003, **24**, 669–681.
16. T. Eskelinen, S. Buss, S. K. Petrovskii, E. V. Grachova, M. Krause, L. Kletsch, A. Klein, C. A. Strassert, I. O. Koshevoy, P. Hirva, *Inorg. Chem.*, 2021, **60**, 8777−8789.
17. V. Sivchik, A. Kochetov, T. Eskelinen, K. S. Kisel, A. I. Solomatina, E. V. Grachova, S. P. Tunik, P. Hirva, I. O. Koshevoy, *Chem. Eur. J.*, 2021, **27**, 1787–1794.
